# Estimating the respiratory syncytial virus-associated hospitalisation burden in older adults in European countries: a systematic analysis

**DOI:** 10.1186/s12916-025-04249-x

**Published:** 2025-08-04

**Authors:** Tiantian Zhang, Rachel M. Reeves, Shihao Ma, Yumeng Miao, Shiqi Sun, Alejandro Orrico-Sánchez, Marcus Panning, Arantxa Urchueguía-Fornes, Danielle Vuichard-Gysin, Harish Nair, Maria João Fonseca, Alen Marijam, Xin Wang, You Li, Respiratory Virus Global Epidemiology Network, Tiantian Zhang, Rachel M. Reeves, Shihao Ma, Yumeng Miao, Shiqi Sun, Alejandro Orrico-Sánchez, Marcus Panning, Arantxa Urchueguía-Fornes, Danielle Vuichard-Gysin, Harish Nair, Maria João Fonseca, Alen Marijam, Xin Wang, You Li, Javier Díez-Domingo, Beatriz Mengual-Chuliá

**Affiliations:** 1 Department of Epidemiology, National Vaccine Innovation Platform, School of Public Health, Nanjing Medical University, Nanjing, China; 2 GSK, Philadelphia, US; 3 Vaccine Research Department, Fisabio-Public Health, Valencia, Spain; 4 CIBERESP, Instituto de Salud Carlos III, Madrid, Spain; 5 Catholic University of Valencia, Valencia, Spain; 6 Institute of Virology, Medical Center University of Freiburg, Germany; Faculty of Medicine, University of Freiburg, Freiburg, Germany; 7 Division of Infectious Diseases and Infection Prevention, Thurgau Hospital Group, Münsterlingen and Frauenfeld, Switzerland; 8 Department of Biostatistics, National Vaccine Innovation Platform, School of Public Health, Nanjing Medical University, Nanjing, China; 9 GSK, Lisbon, Portugal; 10 GSK, Munich, Germany; 11 Centre for Global Health, Usher Institute, University of Edinburgh, Edinburgh, UK; 12 Changzhou Third People’s Hospital, Changzhou Medical Center, Nanjing Medical University, Changzhou, China

**Keywords:** Respiratory syncytial virus, Hospitalisation, Burden of disease, Older adults

## Abstract

**Background:**

With respiratory syncytial virus vaccines recently approved for use among older adults, country-level respiratory syncytial virus (RSV) disease burden estimates are needed to inform local RSV immunisation strategy. We aimed to estimate country-level RSV hospitalisation burden in older adults in Europe.

**Methods:**

We compiled data on RSV hospitalisation burden in adults aged ≥ 60 years in Europe from published studies (systematic review: PROSPERO CRD42024516945), surveillance data, and unpublished data from international collaborators. We adjusted for diagnostic testing, clinical specimens, and case definitions through statistical modelling techniques and generated country-level hospitalisation rate estimates; for countries with no available data, we developed an ensemble model to predict RSV hospitalisation rates. We also estimated RSV in-hospital case fatality ratio (hCFR) for countries with available data.

**Results:**

We included 14 studies (3 unpublished studies). The adjusted RSV-associated hospitalisation rates were overall 2.2 to 6.4 times higher than unadjusted estimates. Among 5 countries with available data, adjusted annual RSV hospitalisation rates ranged from 193/100,000 person-years in the Netherlands (95% confidence interval [CI]: 125–304) and Finland (141–274) to 414/100,000 in Denmark (322–514). The RSV hospitalisation rates predicted by the ensemble model in 23 additional countries ranged from 223/100,000 to 317/100,000 person-years. RSV hCFR ranged from 6.73% (4.63–9.69) in Spain to 10.14% (4.91–19.79) in Switzerland.

**Conclusions:**

This study addresses knowledge gaps in RSV hospitalisation burden among older adults in Europe while highlighting the importance of adjusting for RSV case under-ascertainment. These findings might be relevant for country’s considerations of RSV immunisation strategies for older adults.

**Graphical Abstract:**

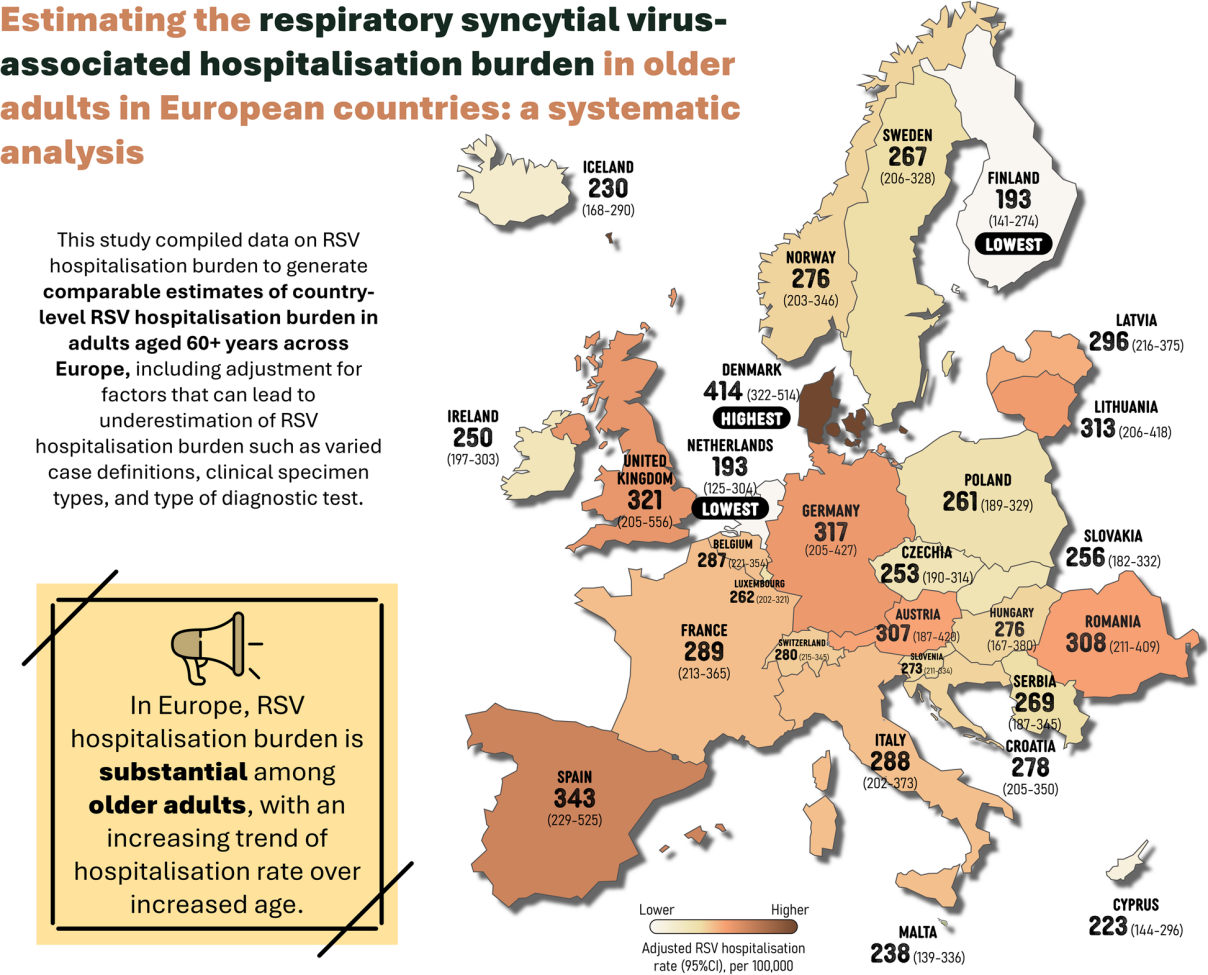

**Supplementary Information:**

The online version contains supplementary material available at 10.1186/s12916-025-04249-x.

## Background

Respiratory syncytial virus (RSV) is a leading cause of acute respiratory infection (ARI), with substantial morbidity and mortality in older adults [1]. A recently published modelling study by Li and colleagues that adjusted for case under-ascertainment related to the use of clinical specimens and diagnostic tests revealed that RSV disease burden is even more substantial than previously estimated: an estimated 787,000 RSV-associated ARI hospitalisations occurred in adults aged 65 years or above in high-income countries in 2019, approximately 2.2 times the unadjusted estimates [2]. Moreover, published systematic literature reviews and meta-analyses show in-hospital case fatality ratio (hCFR) ranged from 6.1% to 8.2% among older adults in high-income countries [2–4].

In 2024, three RSV vaccine candidates (adjuvanted RSVPreF3, GSK; RSVPreF, Pfizer and mRNA-1345, Moderna) were approved for use among older adults [5–7]. European countries such as the UK [8], Germany [9], Denmark [10], Austria [11], Switzerland [12], France [13], Italy [14], Belgium [15] and Sweden [16] have since introduced recommendations for RSV immunisation, while others are considering recommendations. However, data on RSV disease burden among older adults are sparse in Europe; existing studies reporting country-specific estimates have different diagnostic testing, clinical specimens and case definitions, making it impossible to compare RSV disease burden across countries.

This study aimed to improve understanding of RSV hospitalisation burden in older adults at country and regional level across Europe. Addressing data gaps by compiling data from published literature, surveillance and unpublished sources, we consolidated estimates by utilising statistical modelling to adjust for variations in diagnostic testing, clinical specimens and case definitions and used multiple imputation for missing age bands of interest in individual study settings.

## Methods

### Data sources

#### Published data

We conducted a systematic literature review to identify studies reporting RSV disease burden estimates in older adults (defined as 60 years or above) in Europe (registered in PROSPERO, CRD42024516945). We searched PubMed and Embase databases for relevant studies published between 1 January 2000 and 31 December 2023 that reported laboratory-confirmed RSV-associated ARI hospitalisation rate or hCFR among older adults aged 60 years or above in Europe, with no restrictions to case definitions and no language restrictions. Detailed search strategy can be found in Additional file 1: Text S1. Studies that focused only on those with underlying medical conditions were excluded. Considering the ongoing impact of the coronavirus disease (COVID-19) pandemic on RSV disease incidence, we only included RSV hospitalisation data up to the 2019–2020 season (no restrictions for hCFR data). Using a template based on the previous systematic review [2], we collected study-level characteristics (location, country, study period, case definition, clinical specimen, diagnostic test type) and estimates of RSV hospitalisation burden (hospitalisation rate and hCFR) and the corresponding age group. Literature screening and extraction were conducted independently by two groups of reviewers, with disagreements resolved through discussion.

Additionally, we included RSV-associated hospitalisation burden data from the publicly available surveillance report in Denmark [17].

#### Unpublished data from collaborators

We identified research groups that potentially had eligible data on RSV hospitalisation burden through existing collaborators from Respiratory Virus Global Epidemiology Network (RSV GEN) who conducted studies in Europe, studies identified in the aforementioned systematic literature review and studies of influenza hospitalisation burden in adults in Europe identified by a separate review of influenza hospitalisation burden conducted by the study team (details in Additional file 1: Text S2). Research groups willing to contribute unpublished data on RSV hospitalisation burden completed a template with aggregated data on RSV hospitalisation and hCFR for each year of age between 60 and 84 years and 85 years or above as a single age group. Such high granularity in age was used as an important input to help reconcile the reporting variations of age groups in published studies by conducting the age-specific imputation (details in the ‘Data analysis’ section).

### Quality assessment

Both the quality of individual studies (including unpublished studies) and of the corresponding country-level estimates were assessed, independently by two groups of reviewers. For individual studies, we used a modified quality assessment questionnaire based on previous publications [18, 19]. More details on quality assessment can be found in Additional file 1: Table S1 and Table S2.

#### Data analysis

**Fig. 1:**
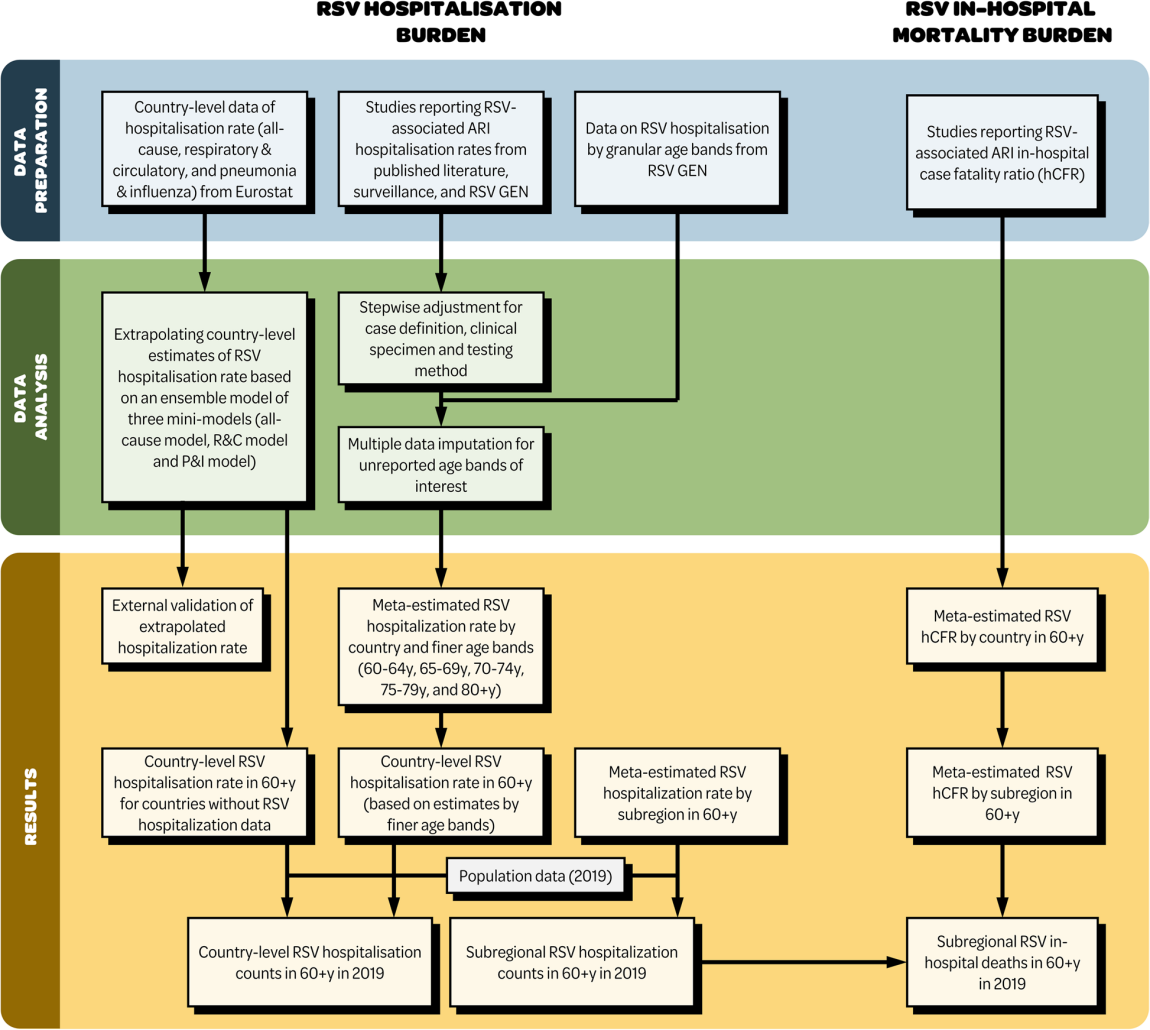
Schematic figure showing the overall study design. RSV respiratory syncytial virus, hCFR in-hospital case fatality ratio, ARI acute respiratory infection, RSV GEN Respiratory Virus Global Epidemiology Network, R&C respiratory and circulatory, P&I pneumonia and influenza, y year

As shown in Fig. 1, we estimated RSV-associated ARI hospitalisation and hCFR burden in older adults aged 60 years or above at the country and region level (according to the Eurostat classification [20]) of Europe.

## RSV-associated ARI hospitalisation burden

### Adjustment for potential RSV under-ascertainment

Previously published studies suggested that RSV could be under-ascertained when a specific clinical specimen and testing approach was used [21, 22]. Therefore, we conducted statistical stepwise adjustments to account for potential underestimation arising from variations in testing methods and clinical specimens. As in our previously published study [2], we defined the combination of polymerase chain reaction (PCR), culture and paired serology as the gold standard testing approach; for clinical specimens, we defined the combination of sputum, serum, saliva and nasopharyngeal specimens as the gold standard respiratory specimens. Moreover, when a study used a case definition narrower than ARI (e.g., influenza-like illness), we conducted additional proportional adjustment.

For each step, we estimated the RSV detection proportion for a specific approach (e.g., PCR), which was ratio of RSV detected by that specific approach to the total RSV detected by the gold standard based on studies that had available data (details in Additional file 1: Table S3 [21, 23–28]); then the reciprocal of the RSV detection proportion was calculated as the adjustment ratio. For the RSV detection proportion, we accounted for the innate statistical uncertainty around the detection proportion through a Bayesian approach (assuming beta-binomial distribution); when more than one study provided data on the detection proportion, a random-effect meta-analysis was conducted to synthesise the detection proportions. We randomly drew 1000 samples from the distribution of the detection proportions above at each step and calculated the products of the adjustment ratios across the three steps as the final adjustment ratio.

### Imputation for missing age groups

We predefined the following finer age bands for estimating and reporting of RSV hospitalisation burden: 60–64 years, 65–69 years, 70–74 years, 75–80 years and 80 years and older. As we anticipated substantial variations in the reporting of age groups in individual studies, a multiple imputation was conducted for studies that did not report all the predefined age bands, similar to the previously published study on burden of RSV in young children [19]. Here, we assumed the increased risk of RSV hospitalisation associated with increased age was mainly due to human-related physiological changes such as immunosenescence [29], rather than external factors such as countries/regions and socioeconomic background; therefore, the imputation was based on the assumption that the hospitalisation rate ratio between two given age bands (e.g., 60–64 years and 65 years or above) was comparable across different studies from different countries (this assumption was verified using data from Spain, Switzerland and Germany, details in Additional file 1: Figure S1). The output of the imputation was 1000 sets of imputed RSV hospitalisation rates for each of the predefined finer age bands per individual study (details in Additional file 1: Text S3).

### Country- and region-level estimation

We generated country-level estimates by conducting a random-effects meta-analysis per set if two or more data points were available for that country. If only one study was available, the estimate from that study was used as the country-level estimate. We used Rubin’s rule [30] to combine 1000 sets of estimates to yield a final estimate of country-specific RSV-associated ARI hospitalisation rate for each finer age group. We estimated the country-level RSV hospitalisation rate and counts in 2019 for 60 years or above (as one single age group), by totalling up the counts of the finer age bands. We further conducted random-effects meta-analysis of the country-level estimates (when there were two or more countries with available estimates) obtained above to estimate RSV hospitalisation rate and counts by region of Europe; when there was only one country for a region, then the estimate for that country was considered the corresponding regional estimate. Using random-effects meta-analysis, we estimated the hospitalisation rate ratio between each of the predefined finer age bands and three broader age groups commonly used in publications (60 years or above, 65 years or above, and 75 years or above), across different individual studies with available data on these finer age bands.

### Extrapolation to countries without RSV hospitalisation burden data

For countries without RSV hospitalisation rate estimates, we conducted extrapolation based on countries with available RSV hospitalisation rate estimates as obtained above using an ensemble model approach that combined the predictions from three individual models. Model 1 assumed RSV hospitalisation rates were proportional to all-cause hospitalisation rate per age band and used country-specific all-cause hospitalisation rate as the predictor from Eurostat; models 2 and 3 replaced all-cause hospitalisation rate with country-level respiratory and circulatory (R&C) hospitalisation and pneumonia and influenza (P&I) hospitalisation rates from Eurostat, respectively. Details of the modelling techniques are available in Additional file 1: Text S4 and Figures S2-4. To further validate the extrapolated estimates, we compared the predicted RSV hospitalisation rates with five external estimates from studies published between January 2024 and April 2025 (not included in this study) [31–35].

## RSV-associated in-hospital case fatality ratio

We estimated country-level RSV-associated ARI hCFR in adults aged 60 years or above, using random-effects meta-analysis for countries with two or more studies. The RSV hCFR was also estimated at the region level of Europe in the same way as the regional estimates of hospitalisation rates; we also estimated the number of RSV-associated ARI in-hospital deaths for any region that had both the estimates of RSV hospitalisation counts and hCFR. In this analysis, we could only estimate hCFR for those tested and found positive for RSV; we did not correct hCFR for under-ascertainment cases as the mortality status for the under-ascertained RSV cases was not known.

As a sensitivity analysis, we excluded data after 2020 as hCFR might be affected by the COVID-19 pandemic. For studies reporting hCFR for 65 years or above (rather than the predefined age group of 60 years or above), we estimated hCFR as an ad hoc analysis.

## Statistical software

All statistical analyses and data visualisations were performed by R version 4.3.2.

## Results

### Study characteristics

We screened a total of 21,196 records from the systematic literature review and included 10 studies [17, 36–44] with eligible data. This was further supplemented by unpublished data from 3 studies shared by international collaborators and a Danish surveillance dataset, totalling 14 included studies. Among these, 9 contributed to the estimate of RSV-associated ARI hospitalisation rate for 5 countries, and 9 contributed to the estimate of RSV-associated ARI hCFR in 9 countries. Detailed information on the included studies can be found in Additional file 1: Figure S5 and Tables S4-5.

### Hospitalisation burden

**Fig. 2:**
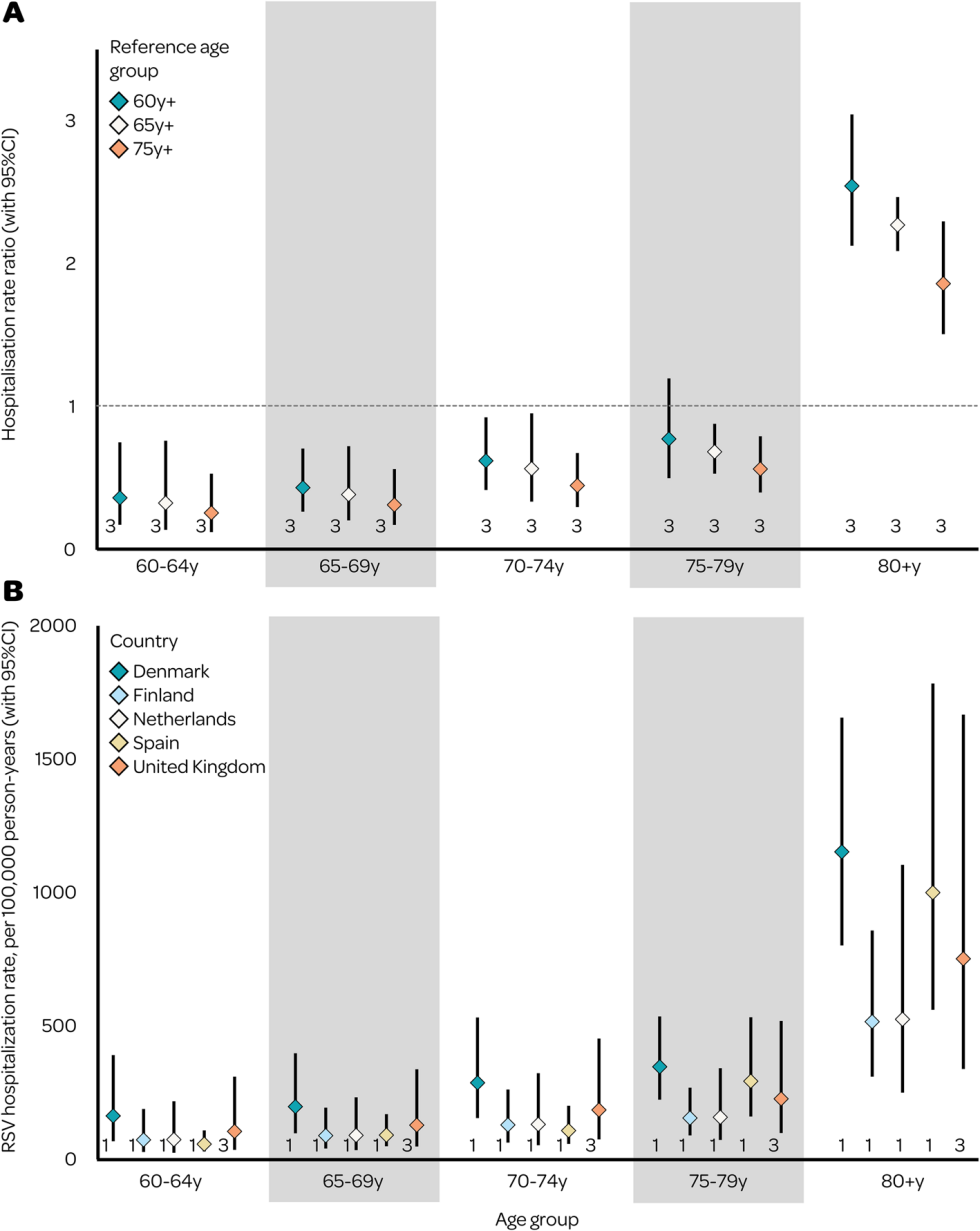
**A** Hospitalisation rate ratio between each finer age band and the reference age groups. **B** Adjusted country-level estimates of RSV-associated ARl hospitalisation rate. The numbers below each line indicate the number of studies. CI=confidence interval, RSV respiratory syncytial virus, y years, ARI acute respiratory infection

We identified five countries (Denmark, Finland, Netherlands, UK, and Spain) with RSV hospitalisation rate data; the unadjusted hospitalisation rate in adults aged 60 years or above ranged from 30/100,000 person-years in Netherlands (95% CI: 26–35) to 188/100,000 (165–215) in Denmark (Table 1). Overall, the RSV-associated ARI hospitalisation rates adjusted for diagnostic testing, clinical specimens and case definitions were approximately 2.2 to 6.4 times higher than unadjusted estimates across countries and age bands (Table 1); adjusted hospitalisation rate ranged from 193/100,000 person-years in Netherlands (95% CI: 125–304) and Finland (95% CI:141–274) to 414/100,000 person-years in Denmark (95% CI: 322–514) (Table 1). Regional estimates informed by the aforementioned 5 countries showed that Southern Europe had the highest RSV hospitalisation rate (343/100,000 person-years, 229–525) and Western Europe had the lowest (246/100,000 person-years, 149–404) in adults aged 60 years or above, despite with overlapping confidence intervals; no studies were available that informed the estimates in Central and Eastern Europe (Table 2). All countries showed an increase in hospitalisation rates with increased age, with a steeper increase observed beyond 75 years of age; the hospitalisation rates for the age groups of 60 years or above were comparable to that for the age band of 75–79 years (Fig. 2).

**Table 1:** Comparison of adjusted and unadjusted RSV-associated ARI hospitalisation rate estimates in adults aged 60 years or above in Europe

Country	Age group	RSV hospitalisation rate, per 100 000 person-years		
		**Adjusted**^a^	**Unadjusted**	**Ratio**
Denmark	60–64 years	163 (68–391)	75 (34–166)	2.17
	65–69 years	198 (98–398)	91 (50–166)	2.18
	70–74 years	287 (155–532)	132 (79–220)	2.17
	75–79 years	347 (224–536)	159 (125–203)	2.18
	≥ 80 years	1153 (802–1656)	529 (485–576)	2.18
	≥ 60 years	414 (322–514)	188 (165–215)	2.20
Finland	60–64 years	73 (28–189)	26 (11–60)	2.81
	65–69 years	89 (41–194)	32 (17–61)	2.78
	70–74 years	129 (63–262)	46 (26–81)	2.80
	75–79 years	155 (90–269)	55 (39–78)	2.82
	≥ 80 years	516 (310–858)	184 (143–238)	2.80
	≥ 60 years	193 (141–274)	68 (56–84)	2.84
Netherlands	60–64 years	74 (25–218)	12 (5–26)	6.17
	65–69 years	90 (35–233)	14 (8–27)	6.43
	70–74 years	131 (53–323)	21 (12–35)	6.24
	75–79 years	158 (73–342)	25 (19–33)	6.32
	≥ 80 years	525 (250–1104)	84 (74–95)	6.25
	≥ 60 years	193 (125–304)	30 (26–35)	6.43
Spain	60–64 years	57 (30–109)	17 (12–23)	3.35
	65–69 years	91 (49–170)	32 (25–40)	3.38
	70–74 years	108 (58–201)	27 (21–34)	3.37
	75–79 years	293 (161–533)	86 (73–102)	3.41
	≥ 80 years	1000 (561–1784)	295 (274–317)	3.39
	≥ 60 years	343 (229–525)	98 (92–104)	3.50
United Kingdom	60–64 years	105 (36–310)	42 (12–144)	2.50
	65–69 years	128 (48–338)	51 (16–159)	2.51
	70–74 years	185 (75–453)	73 (25–217)	2.53
	75–79 years	227 (99–519)	90 (32–253)	2.52
	≥ 80 years	752 (339–1667)	298 (109–815)	2.52
	≥ 60 years	321 (205–556)	130 (74–265)	2.47

*Ratio* ratio of adjusted point estimate to unadjusted point estimate, *RSV* respiratory syncytial virus, *ARI* acute respiratory infection

^a^Adjusted for diagnostic testing, clinical specimens and case definitions used in individual studies

**Table 2:** Country- and region-level estimates of RSV-associated ARI hospitalisation burden in adults aged 60 years or above in Europe

**Country**	**No. studies**	**Data source**	**Years of data**	**Rating**	**Hospitalisation rate (95% CI), per 100,000 person-years**	**Number of hospitalisations in 2019**^a^** (95% CI)**
**Northern Europe**	2	—	—	—	285 (135–603)	73,650 (34,870–155,560)
Denmark	1	Surveillance data [17]	2015–2019	B	414 (322–514)	6170 (5680–6730)
Finland	1	Auvinen et al., 2022 [36]	2016–2020	A	193 (141–274)	3040 (2730–3420)
Iceland	—	Extrapolated	—	—	230 (168–290)	165 (120–208)
Norway	—	Extrapolated	—	—	276 (203–346)	3416 (2504–4278)
Sweden	—	Extrapolated	—	—	267 (206–328)	6992 (5381–8595)
**Central and Eastern Europe**	—	—	—	—	—	—
Croatia	—	Extrapolated	—	—	278 (205–350)	3263 (2407–4106)
Czechia	—	Extrapolated	—	—	253 (190–314)	6979 (5245–8650)
Hungary	—	Extrapolated	—	—	276 (167–380)	7146 (4329–9830)
Latvia	—	Extrapolated	—	—	296 (216–375)	1581 (1157–2006)
Lithuania	—	Extrapolated	—	—	313 (206–418)	2409 (1589–3215)
Poland	—	Extrapolated	—	—	261 (189–329)	25,209 (18,239–31,717)
Romania	—	Extrapolated	—	—	308 (211–409)	15,084 (10,317–20,029)
Serbia	—	Extrapolated	—	—	269 (187–345)	5384 (3753–6925)
Slovakia	—	Extrapolated	—	—	256 (182–332)	3204 (2284–4153)
Slovenia	—	Extrapolated	—	—	273 (211–334)	1533 (1182–1875)
**Western Europe**	4	—	—	—	246 (149–404)	129,550 (78,740–213,140)
Austria	—	Extrapolated	—	—	307 (187–420)	6871 (4180–9395)
Belgium	—	Extrapolated	—	—	287 (221–354)	8354 (6432–10,288)
France	—	Extrapolated	—	—	289 (213–365)	49,768 (36,741–62,994)
Germany	—	Extrapolated	—	—	317 (205–427)	74,943 (48,496–100,886)
Ireland	—	Extrapolated	—	—	250 (197–303)	2372 (1872–2872)
Luxembourg	—	Extrapolated	—	—	262 (202–321)	298 (229–365)
Netherlands	1	Jansen et al., 2007 [41]	1997–2003	C	193 (125–304)	8600 (7460–9950)
Switzerland	—	extrapolated	—	—	280 (215–345)	5890 (4519–7258)
United Kingdom	3	Sharp et al., 2022 [45]	1994–2017	A	321 (205–556)	56,410 (47,350–67,550)
		Mangtani et al., 2006 [43]				
		Fleming et al., 2015 [39]				
**Southern Europe**	1	—	—	—	343 (229–525)	143,170 (95,590–219,140)
Cyprus	—	Extrapolated	—	—	223 (144–296)	523 (338–695)
Italy	—	Extrapolated	—	—	288 (202–373)	50,801 (35,624–65,696)
Malta	—	Extrapolated	—	—	238 (139–336)	298 (174–421)
Spain	1	Orrico-Sánchez, unpublished	2011–2019	B	343 (229–525)	41,260 (35,420–47,310)

Adjusted for diagnostic testing, clinical specimens and case definitions used in individual studies

*CI* confidence interval, *RSV* respiratory syncytial virus, *ARI* acute respiratory infection

^a^Multiplying the hospitalisation rate by the 2019 population from UN [48]

With the extrapolation, RSV hospitalisation rate in adults aged 60 years or above was estimated for an additional 23 countries. In Central and Eastern European countries that did not have available primary RSV hospitalisation burden data, the predicted RSV hospitalisation rate ranged from 253/100,000 person-years (190–314) in Czechia to 313/100,000 person-years (206–418) in Lithuania (Table 2). More detailed results of predicted hospitalisation rates in individual models with comparison to the ensemble model can be found in Additional file 1: Table S6 and Figure S6. External validation results for the ensemble model among four countries showed the extrapolated country-level estimates of RSV hospitalisation rates in adults aged 60 years or above could capture well the variations among countries, with Germany showing a relatively higher hospitalisation rate from both extrapolated and external estimates (Additional file 1: Table S7).

### In-hospital mortality burden

Four countries had available data on RSV-associated ARI hCFR in adults aged 60 years or above: Spain, France, Germany and Switzerland. RSV hCFR ranged from 6.73% (4.63–9.69) in Spain to 10.14% (4.91–19.79) in Switzerland (Table 3). At the regional level, hCFR was 8.72% (8.25–9.22) in Western Europe and was 6.73% (4.63–9.69) in Southern Europe; no estimates were available in Northern or Central and Eastern Europe (Table 3). Seven countries had available data on RSV-associated ARI hCFR in adults aged 65 years or above, ranging from 5.75% (4.28–7.69) in Spain to 12.07% (10.47–13.88) in Denmark (Table 3). The sensitivity analysis that excluded data in 2020 or onwards yielded similar estimates (Additional file 1: Figure S7).

**Table 3:** Country-level estimates of RSV-associated ARI in-hospital case fatality ratio in Europe

Country	No. studies	Data source	Years of data	Rating of estimates	In-hospital CFR (95% CI), %	No. hospitalisation deaths in 2019 (95% CI)
**60 years or above**						
Southern Europe	2	—	—	—	6.73 (4.63–9.69)	11,220 (9570–11,590)
Spain	2	Heppe-Montero et al., 2022 [40]; Orrico-Sánchez, unpublished	2010–2022	A	6.73 (4.63–9.69)	—
Western Europe	3	—	—	—	8.72 (8.25–9.22)	10,190 (8720–10,530)
France	1	Loubet et al., 2024 [42]	2016–2020	C	8.14 (7.64–8.67)	—
Germany	1	Niekler et al., 2023 [44]	2010–2019	C	7.76 (7.11–8.47)	—
Switzerland	1	Chorazka et al., 2021 [38]	2017–2019	B	10.14 (4.91–19.79)	—
**65 years or above**						
Eastern Europe	1	—	—	—	7.69 (2.50–21.30)	—
Czech Republic	1	Beran et al., 2021[23]	2003–2005	C	7.69 (2.50–21.30)	—
Northern Europe^a^	2	—	—	—	9.67 (2.36–39.52)	—
Denmark	1	Surveillance data, 2023 [17]	2015–2018	B	12.07 (10.47–13.88)	—
Southern Europe	2	—	—	—	8.97 (4.07–19.78)	—
Spain	1	Orrico-Sánchez, unpublished	2010–2022	B	5.75 (4.28–7.69)	—
Portugal, Italy, Cyprus	1	Boattini et al., 2021 [37]	2017–2019	C	12.05 (7.91–17.94)	—
Western Europe	1	—	—	—	10.00 (4.56–20.53)	—
Switzerland	1	Chorazka et al., 2021 [38]	2017–2019	B	10.00 (4.56–20.53)	—

*CI* confidence interval, *RSV* respiratory syncytial virus, *ARI* acute respiratory infection, *CFR* in-hospital case fatality ratio

^a^Meta-analysis of data from two countries [17, 36] (Denmark and Finland)

## Discussion

In this study, we compiled a comprehensive dataset of RSV hospitalisation burden in older adults aged 60 years or above in Europe from published studies, surveillance and unpublished data shared by collaborators. We adjusted for diagnostic testing, clinical specimen and case definition through statistical modelling techniques to generate estimates that can be compared among age groups, countries and regions of Europe. For countries without primary data, we developed an ensemble prediction model to estimate RSV hospitalisation rates in 23 additional countries. We also estimated RSV hCFR burden at the country and European region level. This study enhances understanding of the burden of RSV hospitalisations in older adults in Europe and thus provides an important evidence base for national recommendations of RSV immunisation in older adults.

With adjustments for diagnostic testing, clinical specimen and case definition, we estimated that the RSV-associated hospitalisation rate in adults aged 60 years or above ranged from 193/100,000 person-years to 414/100,000 person-years across different countries. These estimates were broadly in line with the recently published RSV-associated hospitalisation rate estimate in 65 years or above in high-income countries, which was 347/100,000 person-years (203–595), using a similar adjustment approach for clinical specimen and diagnostic test [2].

Among four countries with available country-level hCFR estimates in adults aged 60 years or above, RSV hCFR ranged from 6.73% (4.63–9.69) in Spain to 10.14% (4.91–19.79) in Switzerland, suggesting a substantial in-hospital RSV mortality burden in Europe. Among adults aged 65 years or above in seven countries, RSV hCFR estimates ranged from 5.75% to 12.07%, similar to the estimate of 6.1% reported in a recently published meta-analysis among adults aged 65 years or above in high-income countries [2].

Evaluating RSV disease burden in finer age bands within the older adult population is fundamental for determining the age cutoff for RSV immunisation recommendations. However, due to variations in reported age groups in the included studies, comparison of RSV hospitalisation burden across finer age bands was challenging. In this study, we addressed this knowledge gap by requesting RSV hospitalisation data by finer age bands from collaborators. We showed that RSV-associated ARI hospitalisation rate increased with age. Compared to the overall population of 60 years or above, those aged 75 years or above had higher RSV hospitalisation rates, which is also the primary target age group by Joint Committee on Vaccination and Immunisation (JCVI) in the UK [46] and recently by Advisory Committee on Immunization Practices (ACIP) in the USA [47]. Nonetheless, the age bands of 60 to 74 years also had substantially high RSV hospitalisation rates, which were generally comparable to the RSV hospitalisation rate in children aged 1 to < 5 years in high-income countries (170/100,000 person-years) [19], suggesting that expanding immunisation to these age bands could be impactful.

Despite the efforts in collecting unpublished data for this study, this study highlighted remaining data gaps in RSV hospitalisation burden. Regarding RSV hospitalisation rate estimate, only five countries in Europe had available estimates (with statistical uncertainty) in older adults, with no representation from Central and Eastern Europe. This could limit the generalisability of our estimates, particularly the predicted estimates by the extrapolation ensemble model. Nonetheless, external validation of the predicted estimates confirmed the overall good performance of the ensemble model. Regarding RSV hCFR, although more countries had available data (nine countries), some countries had limited sample size for estimating hCFR (e.g., the number of RSV deaths was less than ten in Czech Republic and Switzerland), resulting in wide statistical uncertainty ranges in these countries.

We acknowledge the following limitations. First, most of the country-level estimates were based on only one study and there are variations in hospital-specific contexts such as characteristics of catchment population, admission criteria, and quality of health-care services. To help address this limitation, we used a quality rating system for country-level estimates that incorporated both quantity and quality of the included studies, allowing the readers to interpret the estimates in the context of the overall rating of the estimates. Second, the variations in the predicted country-level RSV hospitalisation rates across countries were smaller than the variations in the estimates based on data, suggesting that the model-predicted estimates could underestimate the differences between countries. In addition, the innate uncertainty around the input data per se further contributed to the uncertainty around the model-predicted estimates. Moreover, as the use of RSV diagnostics among hospitalised patients is dependent on local institutional policies and/or physician discretion, this could have resulted in unmeasured bias regarding the estimation of hCFR. Third, although our adjustment approach improved the disease burden estimation of RSV by accounting for the case under-ascertainment related to the diagnostic testing, clinical specimen and case definition used in the included study, the approach was essentially an approximation of the more realistic disease burden but never the true burden; therefore, we could not rule out overestimation of the disease burden. Due to data scarcity, the parameters used for the adjustment were not restricted to European countries and thus might not fully represent all European countries. Moreover, the case under-ascertainment as discussed in this study was not exclusive to RSV and the adjustment approaches similar to the ones used in our analysis were not widely applied to disease burden estimation studies of other respiratory pathogens (such as influenza); therefore, these results must be interpreted with caution and the RSV disease burden estimates reported in this paper should not be used for direct comparison with unadjusted disease burden estimates of other pathogens. Fourth, for the imputation for unreported age groups, we assumed that the changes in RSV hospitalisation rate over age were due to immunosenescence and that hospitalisation rate ratios between two age groups were identical across different countries. Although our imputation considered the variations in the population age structure, other factors such as health-care seeking behaviour, health system capacity and diagnostic practices were not accounted for in the imputation. Lastly, as the analysis focused on the pre-COVID-19 period, these estimates should be interpreted with caution as RSV epidemiology likely changed during the post COVID-19 pandemic. It is therefore important to establish or strengthen RSV surveillance among older adults at the country level as RSV immunisations are being introduced.

## Conclusions

In summary, this study advances the understanding of country-level RSV hospitalisation burden in older adults in Europe. The resulting estimates could be compared across finer age groups of older adults and different countries or regions of Europe to inform country-specific recommendation of RSV immunisation for older adults and provide important base data for health-economic evaluation of RSV immunisation programmes. This study also identifies key data gaps in country-level RSV hospitalisation burden in older adults and informs the subsequent strengthening of RSV surveillance and RSV research in older adults in Europe.

## Supplementary Information

Additional file 1. Additional details on methods, characteristics of included studies, quality assessment, results of sensitivity and extrapolation. Text S1. Search strategies for the systematic literature review. Text S2. Search strategies for the influenza rapid review. Text S3. Imputation of hospitalisation rates for missing age groups. Text S4. Extrapolation of RSV-associated ARI hospitalisation rate. Table S1. Quality scoring criteria at the individual study level. Table S2. Country-level estimates grading system. Table S3. Adjustment ratios for diagnostic test, clinical specimen and case definition approaches. Table S4. Summary of studies that contributed to RSV-associated ARI hospitalisation rate estimates. Table S5. Summary of studies that contributed to RSV-associated ARI hCFR estimates. Table S6. Model-predicted country-specific RSV-associated ARI hospitalisation rate in adults aged 60 years or above in Europe. Table S7. External validation of extrapolation of RSV-associated ARI hospitalisation ratein adults aged 60 years or above in Europe. Figure S1. Forest plots of hospitalisation rate ratios between each of the five age groups and 65 years above. Figure S2. Correlation of all-cause hospitalisation rate with RSV-associated ARI hospitalisation rate by finer age band. Figure S3. Correlation of respiratory and circulatory hospitalisation rate with RSV-associated ARI hospitalisation rate by finer age band. Figure S4. Correlation of pneumonia and influenza hospitalisation rate with RSV-associated ARI hospitalisation rate by narrower age band. Figure S5. PRISMA flowchart presenting study selection. Figure S6. Model-predicted country-specific RSV-associated ARI hospitalisation rate in adults aged 60 years or above in Europe. Figure S7. Sensitivity analysis results for RSV-associated ARI in-hospital case fatality ratio in adults aged 60 years or above by countries and regions.

## Data Availability

Aggregated data on RSV hospitalisation from published studies are available in GitHub at: https://github.com/TiantianZhang-NJ/RSV_older_Europe. Data from unpublished studies are not publicly available; requests for unpublished data shared by study collaborators should be directly sent to the investigators of the individual studies.

## Abbreviations

ARI: Acute respiratory infection
ACIP: Advisory Committee on Immunization Practices
COVID-19: Coronavirus disease 2019
CI: Confidence interval
hCFR: In-hospital case fatality ratio
JCVI: Joint Committee on Vaccination and Immunisation
PCR: Polymerase chain reaction
P&I: Pneumonia and influenza
RSV: Respiratory syncytial virus
RSV GEN: Respiratory Virus Global Epidemiology Network
R&C: Respiratory and circulatory
