# Supplementary material for: Estimating the respiratory syncytial virus-associated hospitalisation burden in older adults in European countries: a systematic analysis
Source: BMC Med. 2025 Aug 4;23:453. doi: 10.1186/s12916-025-04249-x (PMC12320330; doi:10.1186/s12916-025-04249-x)
Supplement: Supplementary file 1 — Additional file 1. Additional details on methods, characteristics of included studies, quality assessment, results of sensitivity and extrapolation. Text S1. Search strategies for the systematic literature review. Text S2. Search strategies for the influenza rapid review. Text S3. Imputation of hospitalisation rates for missing age groups. Text S4. Extrapolation of RSV-associated ARI hospitalisation rate. Table S1. Quality scoring criteria at the individual study level. Table S2. Country-level estimates grading system. Table S3. Adjustment ratios for diagnostic test, clinical specimen and case definition approaches. Table S4. Summary of studies that contributed to RSV-associated ARI hospitalisation rate estimates. Table S5. Summary of studies that contributed to RSV-associated ARI hCFR estimates. Table S6. Model-predicted country-specific RSV-associated ARI hospitalisation rate in adults aged 60 years or above in Europe. Table S7. External validation of extrapolation of RSV-associated ARI hospitalisation ratein adults aged 60 years or above in Europe. Figure S1. Forest plots of hospitalisation rate ratios between each of the five age groups and 65 years above. Figure S2. Correlation of all-cause hospitalisation rate with RSV-associated ARI hospitalisation rate by finer age band. Figure S3. Correlation of respiratory and circulatory hospitalisation rate with RSV-associated ARI hospitalisation rate by finer age band. Figure S4. Correlation of pneumonia and influenza hospitalisation rate with RSV-associated ARI hospitalisation rate by narrower age band. Figure S5. PRISMA flowchart presenting study selection. Figure S6. Model-predicted country-specific RSV-associated ARI hospitalisation rate in adults aged 60 years or above in Europe. Figure S7. Sensitivity analysis results for RSV-associated ARI in-hospital case fatality ratio in adults aged 60 years or above by countries and regions. [file 12916_2025_4249_MOESM1_ESM.docx]

**Additional file 1**

###### **Manuscript: Estimating the respiratory syncytial virus-associated hospitalisation burden in older adults in European countries: a systematic analysis**

Tiantian Zhang^1^, Rachel M Reeves^2*^, Shihao Ma^1^, Yumeng Miao^1^, Shiqi Sun^1^, Alejandro Orrico-Sánchez^3,4,5^, Marcus Panning^6^, Arantxa Urchueguía-Fornes^3,4^, Danielle Vuichard-Gysin^7^, Harish Nair^8^, Maria João Fonseca^9^, Alen Marijam^10^, Xin Wang^8,11*^, You Li^1,8,12*^, [Respiratory Virus Global Epidemiology Network](https://pubmed.ncbi.nlm.nih.gov/?sort=date&term=Respiratory+Virus+Global+Epidemiology+Network%5BCorporate+Author%5D)

1 Department of Epidemiology, National Vaccine Innovation Platform, School of Public Health, Nanjing Medical University, Nanjing, China

2 GSK, UK

3 Vaccine Research Department, Fisabio-Public Health, Valencia, Spain

4 CIBERESP, Instituto de Salud Carlos III, Madrid, Spain

5 Catholic University of Valencia, Valencia, Spain

6 Institute of Virology, Medical Center University of Freiburg, Germany; Faculty of Medicine, University of Freiburg, Freiburg, Germany

7 Division of Infectious Diseases and Infection Prevention, Thurgau Hospital Group, Münsterlingen and Frauenfeld, Switzerland

8 Centre for Global Health, Usher Institute, University of Edinburgh, Edinburgh, UK

9 GSK, Portugal

10 GSK, Belgium

11 Department of Biostatistics, National Vaccine Innovation Platform, School of Public Health, Nanjing Medical University, Nanjing, China

12 Changzhou Third People's Hospital, Changzhou Medical Center, Nanjing Medical University, Changzhou, China

***Correspondence to**: Prof You Li, Department of Epidemiology, National Vaccine Innovation Platform, School of Public Health, Nanjing Medical University, Nanjing 211166, China You.Li@njmu.edu.cn; Dr. Xin Wang, Department of Biostatistics, National Vaccine Innovation Platform, School of Public Health, Nanjing Medical University, Nanjing 211166, China [Xin.Wang@njmu.edu.cn](mailto:Xin.Wang@njmu.edu.cn); and Dr. Rachel M Reeves ([rachel.m.reeves@gsk.com](mailto:rachel.m.reeves@gsk.com)), GSK London, UK.

Contents

[Supplementary methods 1](#_Toc201075581)

[Search strategy 1](#_Toc201075582)

[Text S1. Search strategies for the systematic literature review 1](#_Toc201075583)

[Text S2. Search strategies for the influenza rapid review 3](#_Toc201075584)

[Quality assessment 4](#_Toc201075585)

[Adjustment for diagnostic test, clinical specimen and case definition 6](#_Toc201075586)

[Text S3. Imputation of hospitalisation rates for missing age groups 7](#_Toc201075587)

[Text S4. Extrapolation of RSV-associated ARI hospitalisation rate 9](#_Toc201075588)

[Supplementary results 12](#_Toc201075589)

[PRISMA flowchart presenting study selection 12](#_Toc201075590)

[Description of individual studies included in the analysis 13](#_Toc201075591)

[Additional results not reported in the main text 18](#_Toc201075592)

[References 23](#_Toc201075593)

List of supplementary tables

[Table S1. Quality scoring criteria at the individual study level 4](#_Toc201052186)

[Table S2. Country-level estimates grading system 5](#_Toc201052187)

[Table S3. Adjustment ratios for diagnostic test, clinical specimen and case definition approaches 6](#_Toc201052188)

[Table S4. Summary of studies that contributed to RSV-associated ARI hospitalisation rate estimates 13](#_Toc201052189)

[Table S5. Summary of studies that contributed to RSV-associated ARI hCFR estimates 16](#_Toc201052190)

[Table S6. Model-predicted country-specific RSV-associated ARI hospitalisation rate in adults aged 60 years or above in Europe 18](#_Toc201052191)

[Table S7. External validation of extrapolation of RSV-associated ARI hospitalisation rate (per 100,000 person-years) in adults aged 60 years or above in Europe 21](#_Toc201052192)

List of supplementary figures

[Figure S1. Forest plots of hospitalisation rate ratios between each of the five age groups and 65 years above 8](#_Toc201052193)

[Figure S2. Correlation of all-cause hospitalisation rate with RSV-associated ARI hospitalisation rate by finer age band (Model 1) 9](#_Toc201052194)

[Figure S3. Correlation of respiratory and circulatory hospitalisation rate with RSV-associated ARI hospitalisation rate by finer age band (Model 2) 10](#_Toc201052195)

[Figure S4. Correlation of pneumonia and influenza hospitalisation rate with RSV-associated ARI hospitalisation rate by narrower age band (Model 3) 11](#_Toc201052196)

[Figure S5. PRISMA flowchart presenting study selection. 12](#_Toc201052197)

[Figure S6. Model-predicted country-specific RSV-associated ARI hospitalisation rate in adults aged 60 years or above in Europe 20](#_Toc201052198)

[Figure S7. Sensitivity analysis results for RSV-associated ARI in-hospital case fatality ratio in adults aged 60 years or above by countries (Panel A) and regions (Panel B) 22](#_Toc201052199)

Supplementary methods

Search strategy

Text S1. Search strategies for the systematic literature review

**PubMed**

*1 RSV*

“Respiratory Syncytial Viruses”[MeSH] OR "Respiratory Syncytial Virus Infections"[Mesh] OR respiratory syncytial[tiab] OR RSV[tiab]

*2 hMPV*

"metapneumovirus"[MeSH Terms] OR "metapneumovirus"[tiab] OR hMPV[tiab]

*3 PIV*

human parainfluenza virus 1[MeSH Terms] OR human parainfluenza virus 2[MeSH Terms] OR human parainfluenza virus 3[MeSH Terms] OR human parainfluenza virus 4[MeSH Terms] OR parainfluenza[tiab] OR PIV[tiab] OR HPIV[tiab]

*4 Measures of frequency*

"Incidence"[Mesh] OR inciden*[tiab] OR "Prevalence"[Mesh] OR prevalen*[tiab] OR frequency[tiab] OR frequencies[tiab] OR rate*[tiab] OR proportion*[tiab] OR distribut*[tiab]

*5 Complications*

“Morbidity”[Mesh] OR morbidity[tiab] OR complication*[tiab] OR "Mortality"[Mesh] OR “Mortality”[subheading] OR mortality[tiab] OR mortalities[tiab] OR "Death"[Mesh] OR death*[tiab] OR case-fatalit*[tiab] OR lethal*[tiab] OR died[tiab] OR "Hospitalization"[Mesh] OR hospital*[tiab] OR "General Practitioners"[Mesh] OR general practitioner[tiab] OR general practitioners[tiab] OR GP[tiab] OR “Delivery of Health Care"[Mesh] OR health care[tiab] OR healthcare[tiab] OR "Ambulatory Care"[Mesh] OR ambulator*[tiab] OR primary care [tiab] OR health resource*[tiab] OR "Critical Care"[Mesh] OR critical care[tiab] OR intensive care[tiab] OR ICU[tiab] OR "Inpatients"[Mesh] OR inpatient*[tiab] OR visit*[tiab] OR consultation*[tiab] OR emergency room[tiab] OR ER[tiab] OR emergency department[tiab] OR ED[tiab]

*6 Risk factors*

caus*[tiab] OR predictor*[tiab] OR determinant*[tiab] OR risk factor*[tiab] OR correlation*[tiab] OR origin*[tiab] OR underlying[tiab]

*7 Animal studies*

Animals[Mesh] NOT (Humans[Mesh] AND Animals[Mesh])

*8 Non-pertinent publication types*

case reports[pt] OR editorial[pt] OR letter[pt] OR news[pt] OR comment[pt]

*9 Inadequate abbreviated terms*

resveratrol[tiab] OR Rous sarcoma virus[tiab] OR Relative search volume[tiab] OR Right subclinical varicocele[tiab] OR Rosuvastatin[tiab] OR Ratio subcutaneous to visceral fat[tiab] OR Rice stripe virus[tiab] OR Recurrent spontaneous vertigo[tiab] OR Retinal slip velocity[tiab] OR Right sinus of valsalva[tiab] OR Mean platelet volume[tiab]

The combination of these search strings (i.e. ((1 OR 2 OR 3) AND (4 OR 5 OR 6)) NOT (7 OR 8 OR 9).

Publications since 2000/01/01 to 2023/12/31

**Embase**

*1 RSV*

'Human respiratory syncytial virus'/exp OR 'respiratory syncytial virus infection'/exp OR “respiratory syncytial”:ti,ab OR RSV:ti,ab

*2 Metapneumovirus*

'Metapneumovirus'/exp OR metapneumovirus:ti,ab OR hMPV:ti,ab

*3 PIV*

‘Parainfluenza’/exp OR ‘PIV’/exp OR ‘HPIV’/exp OR “parainfluenza”:ti,ab OR “PIV”:ti,ab OR “HPIV”:ti,ab

*4 Measures of frequency*

'incidence'/exp OR inciden*:ti,ab OR 'prevalence'/exp OR prevalen*:ti,ab OR frequency:ti,ab OR frequencies:ti,ab OR rate*:ti,ab OR proportion*:ti,ab OR distribut*:ti,ab

*5 Complications*

'morbidity'/exp OR morbidity:ti,ab OR complication*:ti,ab OR 'mortality'/exp OR 'mortality':lnk OR mortality:ti,ab OR mortalities:ti,ab OR 'death'/exp OR death*:ti,ab OR “case-fatalit*”:ti,ab OR lethal*:ti,ab OR died:ti,ab OR 'hospitalization'/exp OR hospital*:ti,ab OR 'general practitioner'/exp OR “general practitioner”:ti:ab OR “general practitioners”:ti:ab OR GP:ti,ab OR ‘Health care delivery’/exp OR “health care”:ti:ab OR healthcare:ti,ab OR 'ambulatory care'/exp OR ambulator*:ti,ab OR “primary care clinic”:ti,ab OR “health resource*”:ti,ab OR ‘Intensive Care’/exp OR “intensive care”:ti:ab OR “critical care”:ti,ab OR ICU:ti:ab OR 'hospital patient'/exp OR inpatient*:ti,ab OR visit*:ti,ab OR consultation*:ti,ab OR “emergency room”:ti,ab OR ER:ti,ab OR “emergency department”:ti,ab OR ED:ti,ab

*6 Risk factors*

caus*:ti,ab OR predictor*:ti,ab OR determinant*:ti,ab OR “risk factor*”:ti,ab OR correlation*:ti,ab OR origin*:ti,ab OR underlying:ti,ab

*7 Animal studies*

'animal'/exp NOT ('human'/exp AND 'animal'/exp)

*8 Pertinent publication types*

[article]/lim OR [article in press]/lim OR [review]/lim

*9 Inadequate abbreviated terms*

resveratrol:ti,ab OR Rous sarcoma virus:ti,ab OR Relative search volume:ti,ab OR Right subclinical varicocele:ti,ab OR Rosuvastatin:ti,ab OR Ratio subcutaneous to visceral fat:ti,ab OR Rice stripe virus:ti,ab OR Recurrent spontaneous vertigo:ti,ab OR Retinal slip velocity:ti,ab OR Right sinus of valsalva:ti,ab OR Mean platelet volume:ti,ab

The combination of these search strings (i.e. ((1 OR 2 OR 3) AND (4 OR 5 OR 6)) NOT (7 OR 8 OR 9).)

Publications since 2000/01/01 to 2023/12/31

Text S2. Search strategies for the influenza rapid review

**Medline**

*1* Influenza*.mp. or exp Influenza, Human/

*2* exp Influenzavirus B/ or exp Influenzavirus A/ or Influenzavirus.mp. or exp Influenzavirus C/

*3* exp Influenza A Virus, H1N1 Subtype/ or Influenza A Virus, H3N2 Subtype/ or pH1N1.mp.

*4* exp Incidence/ or exp Prevalence/ or exp Morbidity/ or morbidity.mp.

*5* exp Hospitalization/ or hospital admission.mp.

*6* exp Mortality/ or exp death/ or mortality.mp.

*7* burden.mp.

*8* exp Europe/ or europe*.mp.

9 (1 or 2 or 3) and [(](#_ENREF_2)4 or 5 or 6 or 7) and 8

limit 9 to (yr="2010 -2022" and "aged (18 plus years)")

**Embase**

*1* Influenza*.mp. or exp Influenza, Human/

*2* exp Influenzavirus B/ or exp Influenzavirus A/ or Influenzavirus.mp. or exp Influenzavirus C/

*3* exp 2009 H1N1 influenza/ or exp "influenza A (H1N1)"/ or exp "influenza A (H3N2)"/ or H1N1pdm.mp.

*4* exp Incidence/ or exp Prevalence/ or exp Morbidity/ or morbidity.mp.

*5* exp Hospitalization/ or hospital admission.mp.

*6* exp Mortality/ or exp death/ or mortality.mp.

*7* burden.mp.

*8* exp Europe/ or europe*.mp.

9 (1 or 2 or 3) and [(](#_ENREF_2)4 or 5 or 6 or 7) and 8

limit 9 to (yr="2010 -2022" and (adult >18 years))

**Web of Science**

*1* TS=(Influenza*) OR TS=(2009 H1N1) OR TS=( H1N1pdm) OR TS=( pH1N1)

*2* TS=(burden) OR TS=(morbidity) OR TS=(Incidence) OR TS=(Prevalence) OR

*3* TS=(Hospitalization) OR TS=(hospital admission) OR TS=(mortality) OR TS=(death)

*4* TS=(europe*)

*5* TS=(adults)

*6* 1 and 2 and 3 and 4 and 5

limit *#*6 to (yr="2010-01-01 – 2022-12-31")

Quality assessment

Table S1. Quality scoring criteria at the individual study level

| Category | Questions | Scoring criteria |
| --- | --- | --- |
| Study design | Q1. Whether cases were prospectively enrolled? | - **Yes –** **1 point** - No/unclear – 0 points |
| Study duration | Q2. Did the study cover three years / seasons or more? | - **Yes – 1 point** - No/unclear – 0 points |
| Study setting | Q3. Whether the study setting represented well the country? | - **Yes (National database / national surveillance network / large-scale multicentre [≥3] study) – 1 point** - No/unclear – 0 points |
| Study subjects | Q4. Any subgroup(s) exclusion that may have affected estimates? | - **No – 1 point** - Yes/unclear – 0 points (e.g. insurance claim database) |
| Case definition | Q5. Whether the case definition was clear and consistently used in the study? | - Yes – **1 point** - No/unclear – 0 points |
| Sampling strategy | Q6. What is the proportion of eligible ALRI cases that were tested for RSV? | - ≥90% – **1 point** - <90% but a systematic sample of eligible cases were tested – **1 point** - <90%/unclear/ecological studies – 0 points |
| Estimation techniques | Q7. Whether appropriate adjustment was made for the estimation | - For studies estimating for incidence / hospitalisation rate, adjustment was made to account for the underlying catchment population of the study site. **– 1 point** - For excess morbidity / mortality modelling studies, adjustment was made to account for the role of other co-circulating pathogens (e.g., influenza) – **1 point** - No adjustments made – 0 points |

ALRI = acute lower respiratory infection; RSV = respiratory syncytial virus.

Table S2. Country-level estimates grading system

| Study-level grade | Total score |
| --- | --- |
| A (very good) | Estimates based on:   - ≥1 study with an overall score of 6-7; OR - ≥2 studies with an overall score of 4-5; OR - 1 study with an overall score of 4-5 and ≥2 studies with an overall score of 2-3 |
| B (good) | Estimates based on:   - 1 study with an overall score of 4-5; OR - ≥2 studies with an overall score of 2-3 |
| C (fair) | Estimates based on:   - ≥1 study with an overall score of 0-3 |

Adjustment for diagnostic test, clinical specimen and case definition

Table S3. Adjustment ratios for diagnostic test, clinical specimen and case definition approaches

| **Candidate approach** | **Gold standard approach** | **Detection proportion**  **posterior distribution** | **Reference** | **Adjustment ratio (95% CI)** |
| --- | --- | --- | --- | --- |
| **Diagnostic test** | | | | |
| PCR | Serology + PCR + Culture | Beta (88, 31) | Falsey et al. 2002[[1](#_ENREF_1)] | 1.35 (1.23–1.52) |
| RAD | PCR | Beta (33,19) | Onwuchekwa et al. 2023[[2](#_ENREF_2)] | —— |
| RAD | Serology + PCR + Culture | Beta (88,31) * Beta (33,19) | Calculated based on above | 2.14 (1.73–2.76) |
| PCR + Culture | Serology + PCR + Culture | Beta (88, 31) | Falsey et al. 2002[[1](#_ENREF_1)] | 1.35 (1.22–1.52) |
| Unclear | Serology + PCR + Culture | Beta (88,31) * Beta (33,19) | Assuming same sensitivity to RAD | 2.13 (1.73–2.72) |
| **Respiratory specimen** |  |  |  |  |
| NPS | Serum + NPS + Saliva + Sputum | Beta (32,20) | Ramirez et al. 2022[[3](#_ENREF_3)] | 1.62 (1.36–2.07) |
| Unclear | Serum + NPS + Saliva + Sputum | Beta (32,20) | Assuming same sensitivity to NPS | 1.63 (1.36–2.09) |
| **Case definition** | | | | |
| ILI | ARI | Beta (14,8) | Hirve et al. 2020[[4](#_ENREF_4)] | 1.56 (1.22–2.27) |
| SARI | ARI | Beta (44,13) | Hirve et al. 2020[[4](#_ENREF_4)] | 1.29 (1.15–1.53) |
| LRI | ARI | Sigmoid (Normal (0.1725,0.2951)) | Papi et al. 2023[[5](#_ENREF_5)]  Walsh et al. 2023[[6](#_ENREF_6)]  Beran et al. 2021[[7](#_ENREF_7)] | 1.86 (1.53–2.35) |

PCR = polymerase chain reaction. RAD = rapid antigen detection. NPS = nasopharyngeal swab. ARI = acute respiratory infection. ILI = influenza-like illness. LRI = lower respiratory infection. SARI = severe acute respiratory infection. CI = confidence interval.

Text S3. Imputation of hospitalisation rates for missing age groups

For individual studies that did not report all of the predefined age bands (60–64 years, 65–69 years, 70–74 years, 75–80 years, and 80 years), we conducted imputation for the missing age groups based on the hospitalisation rate ratio between the reported age group in those studies and the targeted (i.e., missing) age group.

First, we calculated the hospitalisation rate ratio for the reported age group versus the targeted age group for studies that had available rate ratio data between the two age groups and then synthesised the rate ratios by random-effects meta-analysis, which resulted in a pooled estimate (with standard error) of the rate ratio between the two age groups.

Subsequently, we generated 1000 samples of hospitalisation rate ratio from a normal distribution of log hospitalisation rate ratio and its standard errors, and applied these samples to the hospitalisation rates for the reported age group to obtain the 1000 imputed hospitalisation rates for the targeted age group. By repeating the exercise for all studies that need imputation, a total of 1000 imputed datasets were generated, representing the uncertainty around the imputation.

Finally, for each of the 1000 imputed datasets, meta-analysis was conducted (together with studies that did not require imputation) and the resulting 1000 sets of pooled estimates (with standard errors) was combined following Rubin’s rule [[8](#_ENREF_8)].


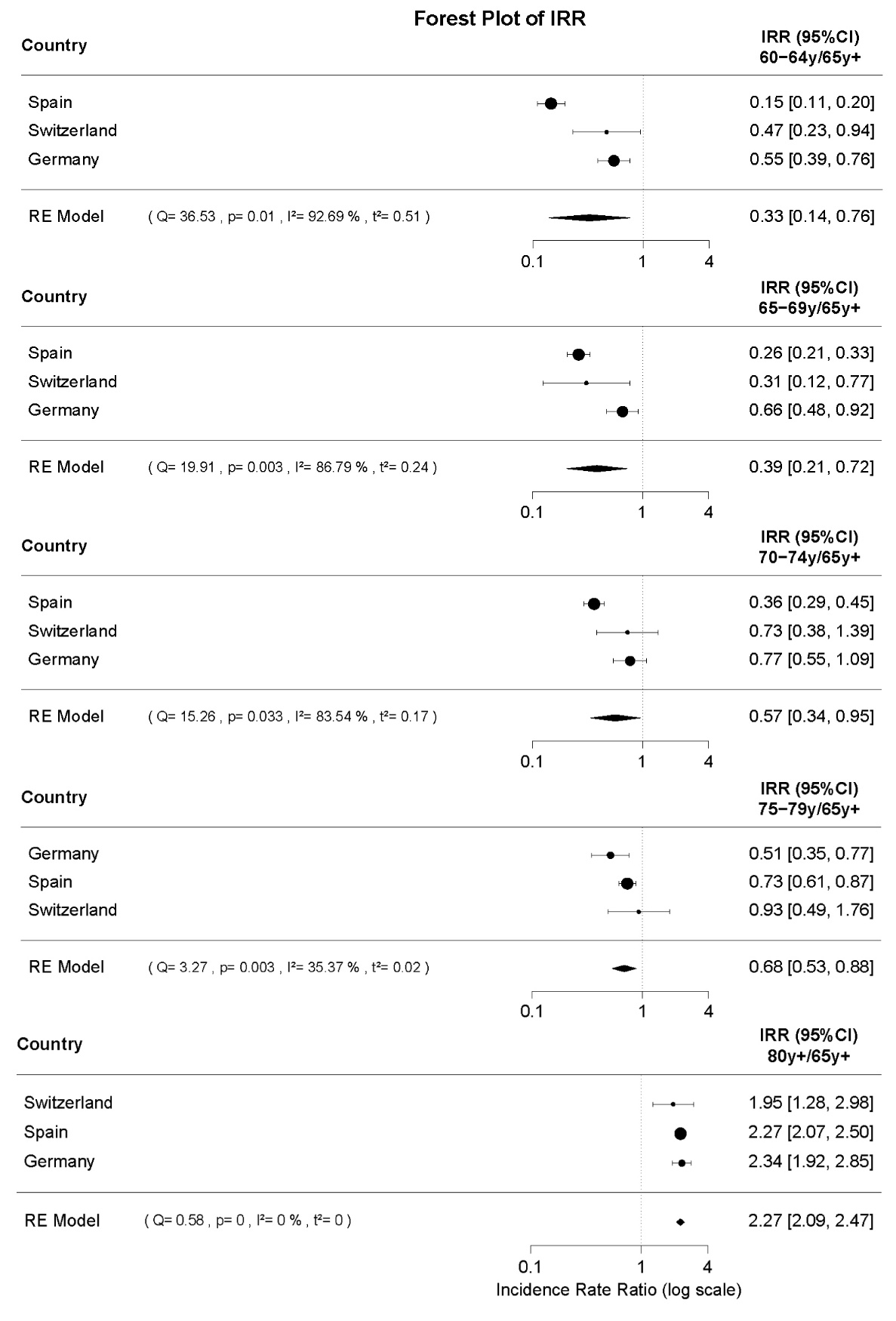


Figure S1. Forest plots of hospitalisation rate ratios between each of the five age groups and 65 years above

IRR = hospitalisation rate ratios. CI = Confidence Interval. RE = random effects.

Text S4. Extrapolation of RSV-associated ARI hospitalisation rate

We used country-specific all-cause hospitalisation rates (all-cause), respiratory and circulatory (R&C) hospitalisation rates, and pneumonia and influenza (P&I) hospitalisation rates from Eurostat as predictors, separately as Model 1, Model 2, and Model 3. Where available, we used the data for the predictors for the median year of the corresponding respiratory syncytial virus hospitalisation rate data in each country; otherwise, the year closest to the median year was selected. Linear regression analysis was conducted for each model and each finer age band (i.e., 60-64 years, 65-69 years, 70-74 years, 75-79 years and 80 years or above). The detailed results on the correlation between predictors and RSV hospitalisation rates are presented below, for the three models.

For each model, we generated a set of 1000 predictive RSV hospitalisation rates and counts for each finer age band; these set of 1000 counts were then totalled up across different finer age bands, yielding a set of 1000 RSV hospitalisation counts and rates for the age group of 60 years or above. Next, we pooled up the RSV hospitalisation rates for 60 years or above generated from the three models (a total of 1000$\times$3 = 3000 predictive rates) and calculated the median, 2.5^th^ percentile and 97.5^th^ percentile of the rates as the point estimate and 95% predictive intervals of the predicted RSV hospitalisation rates.


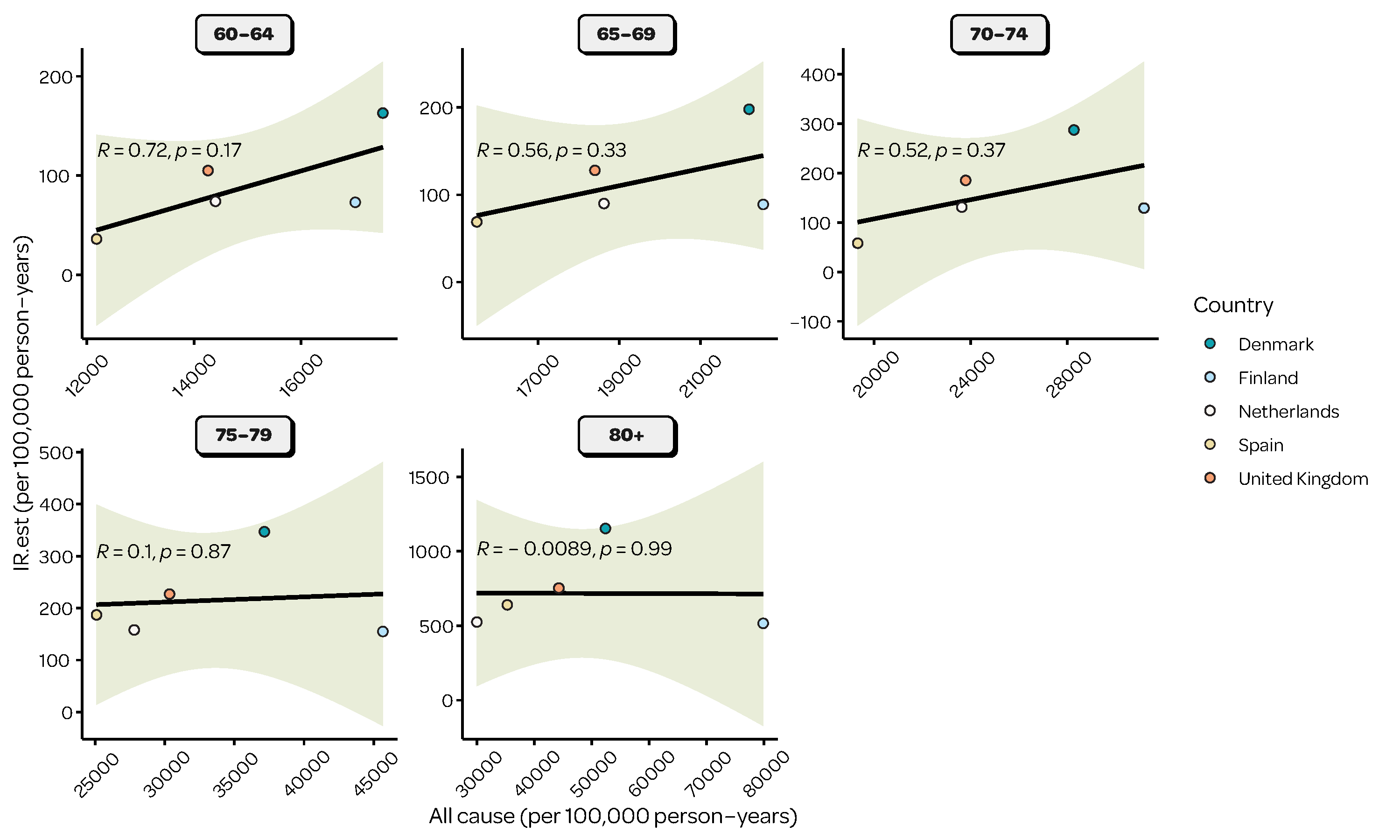


Figure S2. Correlation of all-cause hospitalisation rate with RSV-associated ARI hospitalisation rate by finer age band (Model 1)

RSV = respiratory syncytial virus. ARI = acute respiratory infection. Allcause = all-cause hospitalisation rate. IR.est = RSV-associated ARI hospitalisation rate. R = Correlation Coefficient.


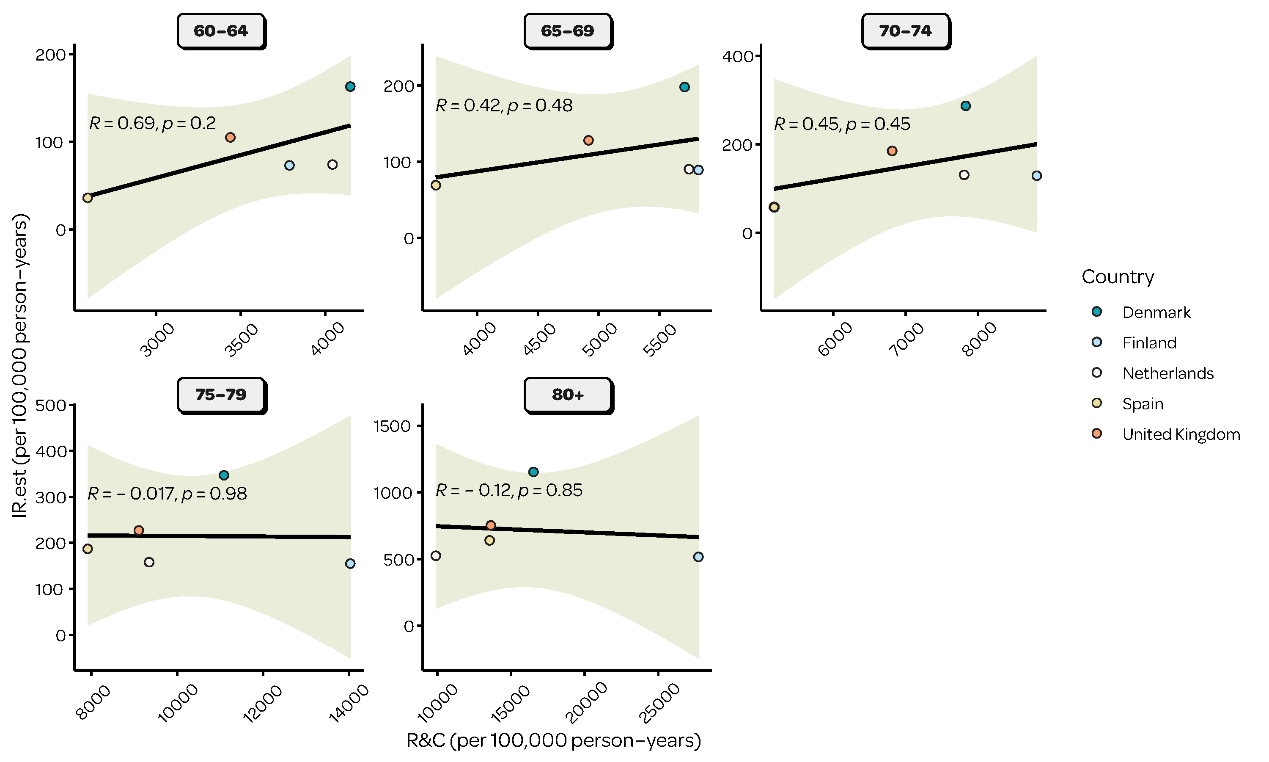


Figure S3. Correlation of respiratory and circulatory hospitalisation rate with RSV-associated ARI hospitalisation rate by finer age band (Model 2)

RSV = respiratory syncytial virus. ARI = acute respiratory infection. R&C = respiratory and circulatory hospitalisation rate. IR.est = RSV-associated ARI hospitalisation rate. R = Correlation Coefficient.


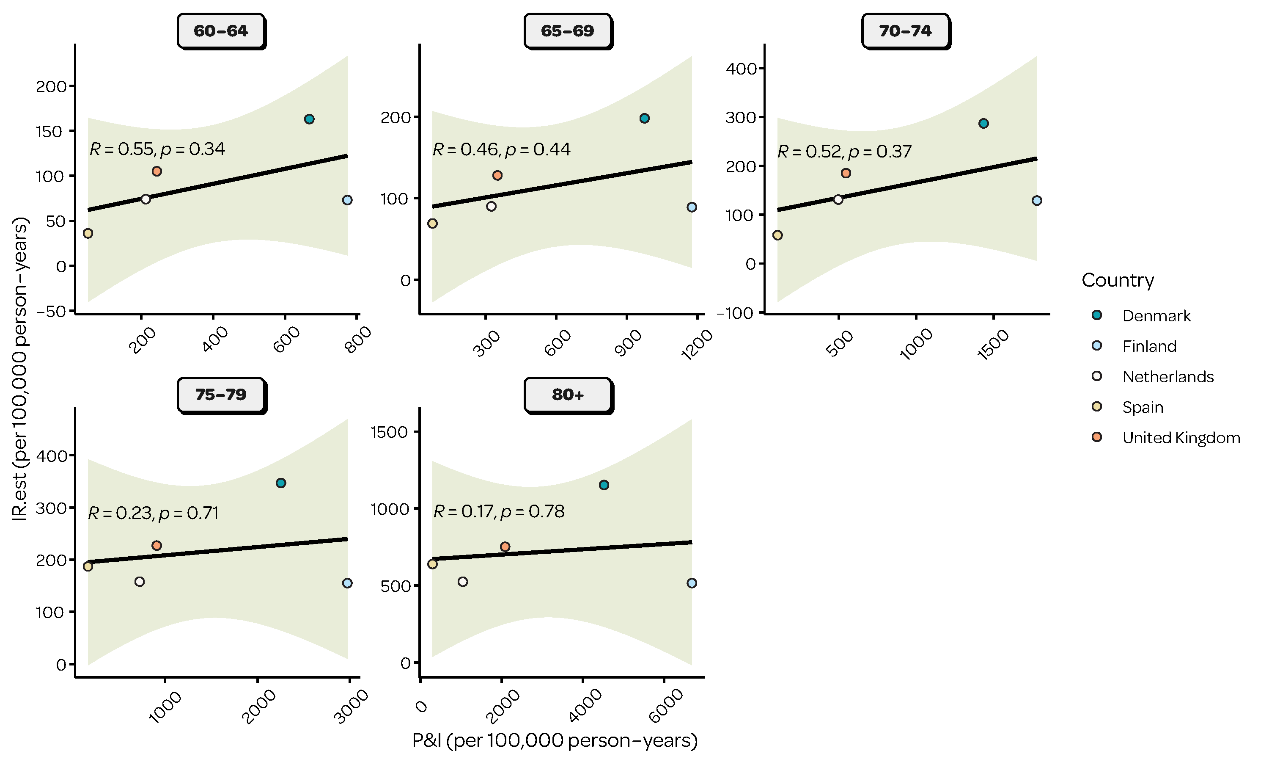


Figure S4. Correlation of pneumonia and influenza hospitalisation rate with RSV-associated ARI hospitalisation rate by narrower age band (Model 3)

RSV = respiratory syncytial virus. ARI = acute respiratory infection. P&I = pneumonia and influenza hospitalisation rate. IR.est = RSV-associated ARI hospitalisation rate. R = Correlation Coefficient.

Supplementary results

PRISMA flowchart presenting study selection


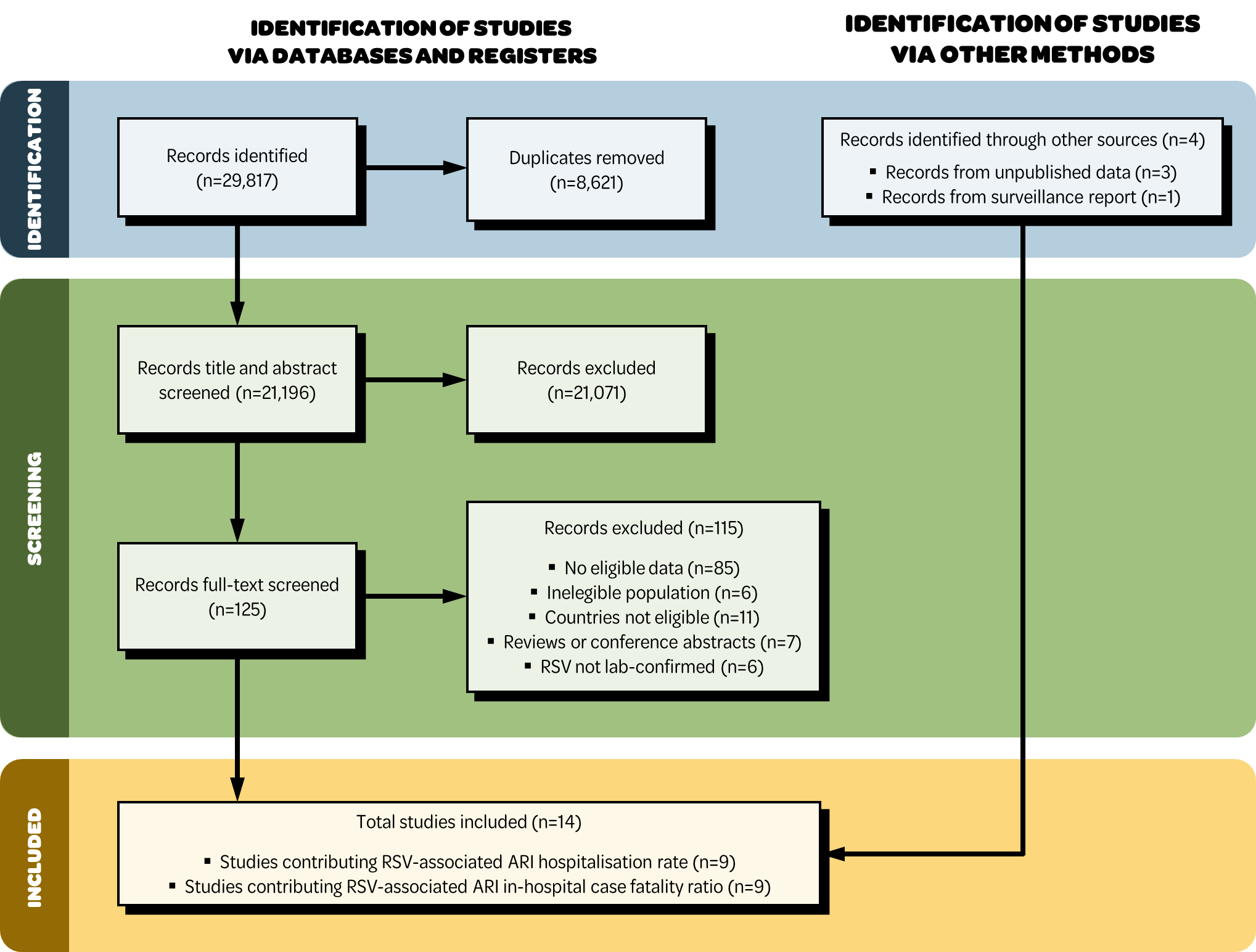


Figure S5. PRISMA flowchart presenting study selection.

RSV=respiratory syncytial virus. ARI=acute respiratory infection. n= number.

Description of individual studies included in the analysis

Table S4. Summary of studies that contributed to RSV-associated ARI hospitalisation rate estimates

|  |  |  |  |  |  |  |  | **Outcome reported by age groups** | | | | | | |  |
| --- | --- | --- | --- | --- | --- | --- | --- | --- | --- | --- | --- | --- | --- | --- | --- |
| **Data source** | **Description of data source** | **Country** | **Time period** | **Sample size (no. of RSV)** | **Case definition** | **Clinical specimen** | **Diagnostic test** | **60+** | **60–64** | **65-69** | **70-74** | **75-79** | **80+** | **Others** | **Used for age group imputation*** |
| Sharp et al., 2022[[9](#_ENREF_9)] | Retrospective excess hospitalization modelling analysis | United Kingdom | 2010/08-2017/07 | 14,373 (modelled) | ARI | NPS | PCR | No | No | No | No | No | No | 65-74,  75+ | No |
| Jansen et al., 2007[[10](#_ENREF_10)] | Retrospective excess hospitalization modelling analysis | Netherlands | 1997–2003 | NA (modelling analysis with no relevant information) | LRI | Unclear | RAD | No | No | No | No | No | No | 65+ | No |
| Mangtani et al., 2006[[11](#_ENREF_11)] | Retrospective excess hospitalization modelling analysis | United Kingdom | 1994/04-2000/03 | NA (modelling analysis with no relevant information) | ARI | Unclear | Unclear | No | No | No | No | No | No | 65+ | No |
| Auvinen et al., 2022[[12](#_ENREF_12)] | Retrospective hospital-based cohort analysis | Finland | 2016/11-2020/05 | 106 | SARI | NPS | PCR | No | No | No | No | No | No | 65+ | No |
| Fleming et al., 2015[[13](#_ENREF_13)] | Retrospective excess hospitalization modelling analysis | United Kingdom | 1995-2009 | 14,039 (modelled) | ARI | Unclear | PCR,  Culture | No | No | No | No | No | No | 65+ | No |
| Panning, Unpublished data | Retrospective hospital database analysis | Germany | 2018/01-2023/06 | 319 | ARI | NPS | PCR | Yes | Yes | Yes | Yes | Yes | Yes | NA | Yes |
| Orrico-Sánchez, Unpublished data | Retrospective hospital database analysis | Spain | 2010/11 - 2019/11 | 1034 | ILI | NPS | PCR | Yes | Yes | Yes | Yes | Yes | Yes | NA | Yes |
| Statens Serum Institut[[14](#_ENREF_14)] | Retrospective hospital database analysis | Denmark | 2015/2016-2018/2019 | 8393 | ARI | Unclear | PCR | No | No | No | No | No | No | 65-74,  75-84,  85+ | No |
| Chorazka et al., 2021[[15](#_ENREF_15)], and unpublished data† | Retrospective multi-center hospital database analysis | Switzerland | 2017/10-2019/04 | 69 | ARI | NPS | PCR | Yes | Yes | Yes | Yes | Yes | Yes | NA | Yes |

*Referring to studies that had available data on RSV hospitalisation counts by granular age band (by each year of age); these data were used for imputation for studies that did not report all of the prespecified age groups of interest. †These studies did not contribute directly any RSV-associated ARI hospitalisation rates but the RSV hospitalisation counts by granular age band. RSV=respiratory syncytial virus. ARI=acute respiratory infection. LRI=lower respiratory tract infection. SARI=severe acute respiratory infection. NPS=nasopharyngeal swab. PCR=polymerase chain reaction. Culture=viral culture. ILI=Influenza-like-illness. RAD=rapid antigen detection. NA=not available.

Table S5. Summary of studies that contributed to RSV-associated ARI hCFR estimates

|  |  |  |  |  |  |  |  | **Outcome reported by age groups** | | | | | | |  |
| --- | --- | --- | --- | --- | --- | --- | --- | --- | --- | --- | --- | --- | --- | --- | --- |
| **Data source** | **Description of data source** | **Country** | **Time period** | **Sample size (no. of RSV)** | **Case definition** | **Clinical specimen** | **Diagnostic test** | **60+** | **60–64** | **65-69** | **70-74** | **75-79** | **80+** | **Others** |  |
| Chorazka et al., 2021[[15](#_ENREF_15)], with unpublished data | Retrospective multi-center hospital database analysis | Switzerland | 2017/10-2019/04 | 69 | ARI | NPS | PCR | Yes | Yes | Yes | Yes | Yes | Yes | NA |  |
| Auvinen et al., 2022[[12](#_ENREF_12)] | Prospective hospital - based cohort | Finland | 2018-2020 | 22 | SARI | NPS | PCR | No | No | No | No | No | No | 65+ |  |
| Beran et al., 2021[[7](#_ENREF_7)] | Prospective long term care facilities - based | Czechia | 2003/10-2005/04 | 39 | ARI | NPS | PCR | No | No | No | No | No | No | 65+ |  |
| Boattini et al., 2021[[16](#_ENREF_16)] | Retrospective hospital database analysis | Portugal, Italy, Cyprus | 2017/10-2019/04 | 166 | LRI | NPS | PCR | No | No | No | No | No | No | 65+ | |
| Orrico-Sánchez, Unpublished data | Retrospective hospital database analysis | Spain | 2010/11 - 2022/11 | 778 | ILI | NPS | PCR | Yes | Yes | Yes | Yes | Yes | Yes | NA |  |
| Statens Serum Institut [[14](#_ENREF_14)] | Retrospective hospital database analysis | Denmark | 2015/2016-2018/2019 | 1408 | ARI | Unclear | PCR | No | No | No | No | No | No | 65-74,  75-84,  85+ |  |
| Heppe-Montero et al., 2022[[17](#_ENREF_17)] | Retrospective hospital database analysis | Spain | 2012-2020 | 17312 | LRI | Unclear | Unclear | Yes | No | No | No | No | No | NA |  |
| Niekler et al., 2023[[18](#_ENREF_18)] | Retrospective hospital database analysis | Germany | 2010-2019 | 5900 | ARI | Unclear | Unclear | Yes | No | No | No | No | No | NA |  |
| Loubet et al., 2024[[19](#_ENREF_19)] | Retrospective hospital database analysis | France | 2016-2020 | 10810 | ARI | Unclear | Unclear | Yes | No | No | No | No | No | NA |  |

RSV=respiratory syncytial virus. ARI=acute respiratory infection. SARI=severe acute respiratory infection. NPS=nasopharyngeal swab. PCR=polymerase chain reaction. LRI = lower respiratory tract infection. ILI=Influenza-like-illness. NA=not available. hCFR=in-hospital case fatality ratio.

Additional results not reported in the main text

Table S6. Model-predicted country-specific RSV-associated ARI hospitalisation rate in adults aged 60 years or above in Europe

| **Region** | **Country** | **Predicted RSV hospitalisation rate (95% CI), per 100 000 person-years** | | | |
| --- | --- | --- | --- | --- | --- |
|  |  | **Model 1**  **(all-cause hospitalisation)** | **Model 2**  **(R&C hospitalisation)** | **Model 3**  **(P&I hospitalisation)** | **Ensemble model** |
| **Northern Europe** | Denmark | 268 (210–323) | 262 (207–316) | 260 (197–321) | 263 (205–320) |
|  | Finland | 261 (146–376) | 247 (130–364) | 286 (186–387) | 265 (154–375) |
|  | Iceland | 232 (171–291) | 235 (161–307) | 221 (168–274) | 230 (168–290) |
|  | Norway | 284 (212–351) | 272 (191–346) | 273 (204–339) | 276 (203–346) |
|  | Sweden | 269 (206–331) | 268 (206–330) | 265 (205–324) | 267 (206–328) |
| **Central and Eastern Europe** | Croatia | 304 (223–384) | 289 (214–366) | 242 (181–301) | 278 (205–350) |
|  | Czechia | 282 (210–351) | 258 (189–324) | 221 (172–267) | 253 (190–314) |
|  | Hungary | 290 (120–459) | 302 (196–416) | 232 (182–283) | 276 (167–380) |
|  | Latvia | 312 (240–384) | 308 (201–413) | 267 (202–332) | 296 (216–375) |
|  | Lithuania | 346 (249–441) | 317 (153–479) | 277 (213–340) | 313 (206–418) |
|  | North Macedonia | 223 (160–284) | - | - | - |
|  | Poland | 294 (219–366) | 279 (200–360) | 210 (149–271) | 261 (189–329) |
|  | Romania | 354 (237–477) | 321 (195–456) | 247 (188–307) | 308 (211–409) |
|  | Serbia | 298 (207–384) | 286 (202–369) | 219 (153–285) | 269 (187–345) |
|  | Slovakia | 287 (204–373) | 266 (165–371) | 214 (169–258) | 256 (182–332) |
|  | Slovenia | 287 (219–353) | 280 (218–342) | 252 (196–308) | 273 (211–334) |
| **Western Europe** | Austria | 353 (183–521) | 303 (172–429) | 265 (205–324) | 307 (187–420) |
|  | Belgium | 309 (239–380) | 297 (229–364) | 257 (194–321) | 287 (221–354) |
|  | France | 309 (215–401) | 296 (228–366) | 262 (196–328) | 289 (213–365) |
|  | Germany | 360 (215–501) | 324 (192–452) | 271 (204–338) | 317 (205–427) |
|  | Ireland | 253 (201–306) | 257 (201–311) | 240 (189–290) | 250 (197–303) |
|  | Luxembourg | 279 (217–340) | 269 (209–328) | 238 (180–297) | 262 (202–321) |
|  | Netherlands | 242 (155–326) | 258 (179–333) | 227 (169–285) | 242 (167–315) |
|  | Switzerland | 301 (225–374) | 284 (221–347) | 256 (196–315) | 280 (215–345) |
|  | United Kingdom | 260 (197–323) | 270 (207–332) | 273 (200–344) | 267 (201–333) |
| **Southern Europe** | Cyprus | 225 (143–302) | 243 (156–320) | 200 (132–267) | 223 (144–296) |
|  | Italy | 299 (198–398) | 304 (225–383) | 261 (183–338) | 288 (202–373) |
|  | Malta | 230 (43–416) | 249 (191–309) | 234 (182–285) | 238 (139–336) |
|  | Spain | 287 (200–375) | 288 (214–361) | 263 (194–331) | 279 (203–355) |

RSV = respiratory syncytial virus. ARI = acute respiratory infection. CI = confidence interval. R&C = respiratory and circulatory. P&I = pneumonia and influenza (P&I). Ensemble = combining the estimates of models 1–3.


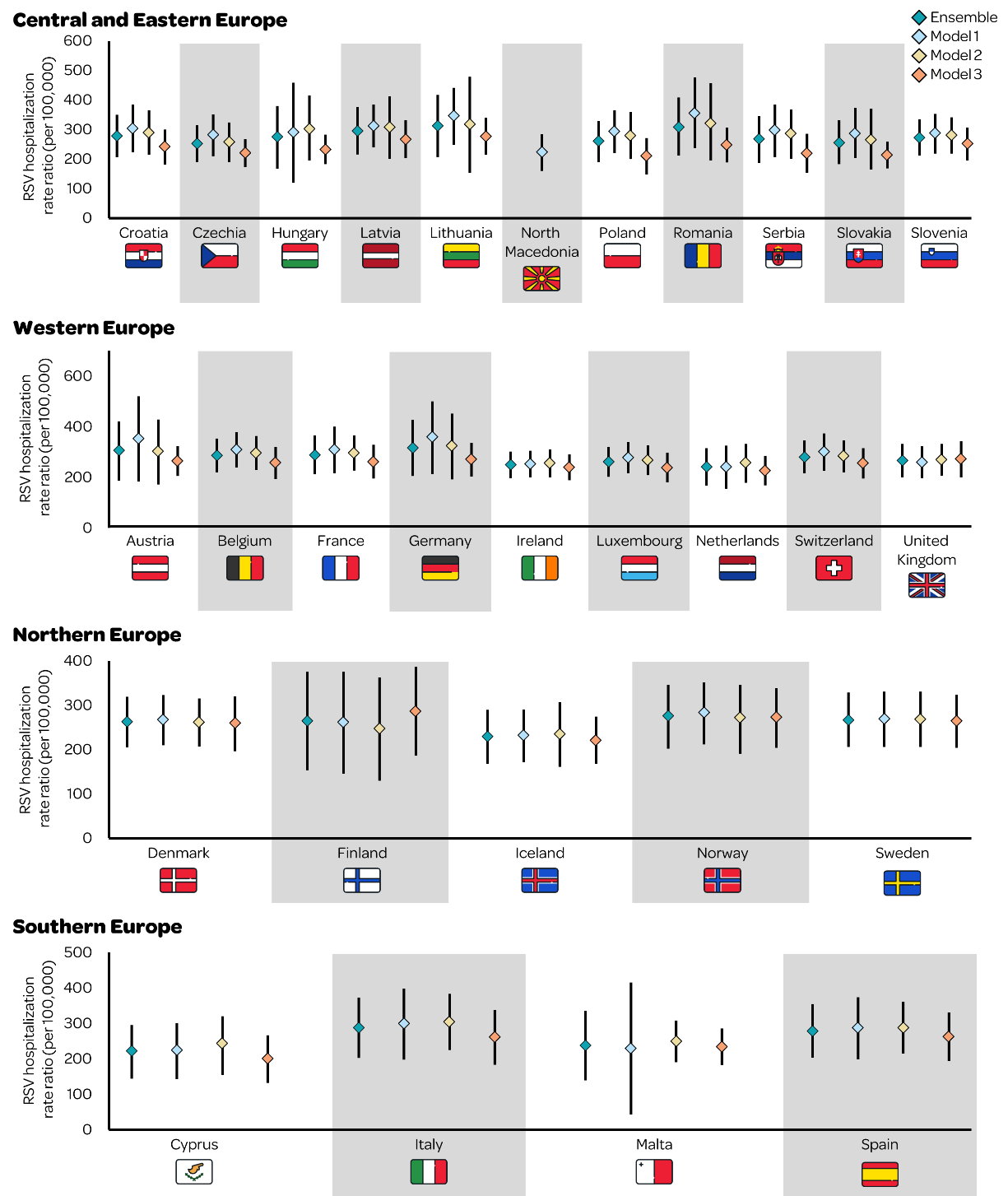


Figure S6. Model-predicted country-specific RSV-associated ARI hospitalisation rate in adults aged 60 years or above in Europe

Model 1 = RSV-associated ARI hospitalisation rate predicted by all-cause hospitalisation rate as a predictor. Model 2 = RSV-associated ARI hospitalisation rate predicted by respiratory and circulatory hospitalisation rate as a predictor. Model 3 = RSV-associated ARI hospitalisation rate predicted by pneumonia and influenza hospitalisation rate as a predictor. Ensemble = combining the estimates of models 1–3. RSV = respiratory syncytial virus. ARI = acute respiratory infection.

Table S7. External validation of extrapolation of RSV-associated ARI hospitalisation rate (per 100,000 person-years) in adults aged 60 years or above in Europe

| **Country** | **Extrapolated from this analysis (95% CI)** | **External estimates and remarks** |
| --- | --- | --- |
| Spain | 279 (203–355) | 257–283[[20](#_ENREF_20)];  (the range of estimates in 2016–2019) |
| Germany | 317 (205–427) | 236–363[[21](#_ENREF_21)];  (the range of estimates in 2015–2019) |
|  |  | 402 (261–609)[[22](#_ENREF_22)] |
| Denmark | 263 (205–320) | 258–352[[23](#_ENREF_23)];  (the range of estimates for 55 years or above and for 65 years or above) |
| Italy | 288 (202–373) | 223 (135–303)[[24](#_ENREF_24)] |

CI=Confidence Interval. RSV=respiratory syncytial virus. ARI=acute respiratory infection.


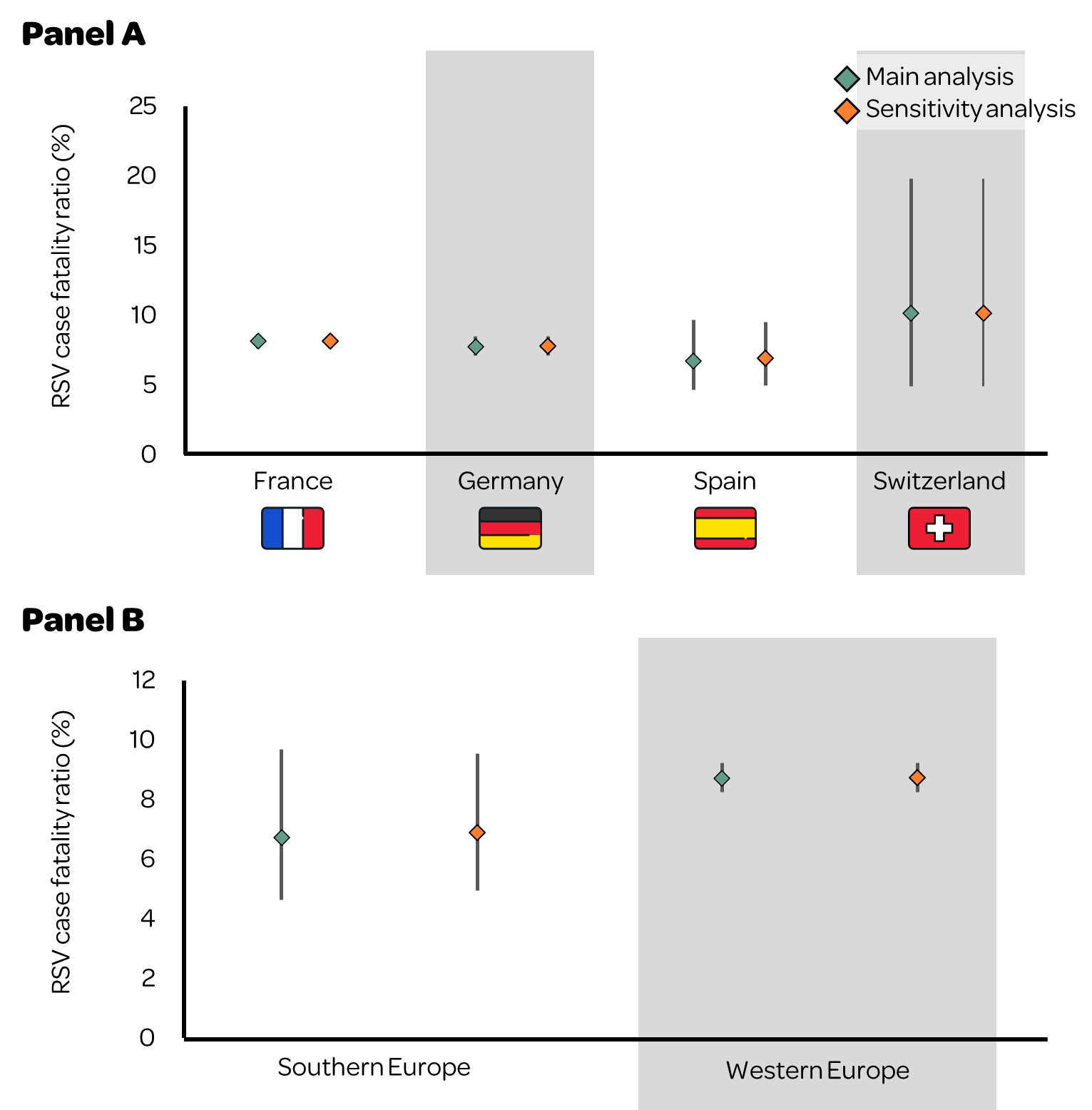


Figure S7. Sensitivity analysis results for RSV-associated ARI in-hospital case fatality ratio in adults aged 60 years or above by countries (Panel A) and regions (Panel B).

RSV = respiratory syncytial virus. ARI = acute respiratory infection.

# References

1. Falsey AR, Formica MA, Walsh EE. Diagnosis of respiratory syncytial virus infection: comparison of reverse transcription-PCR to viral culture and serology in adults with respiratory illness. J Clin Microbiol, 2002; 40(3):817-820.

2. Onwuchekwa C, Moreo LM, Menon S, Machado B, Curcio D, Kalina W, et al. Underascertainment of Respiratory Syncytial Virus Infection in Adults Due to Diagnostic Testing Limitations: A Systematic Literature Review and Meta-analysis. J Infect Dis, 2023; 228(2):173-184.

3. Ramirez JA, Carrico R, Wilde AM, Junkins A, Furmanek S, Chandler TR, et al. 371. Adding sputum and saliva to nasopharyngeal swab samples for PCR detection of Respiratory Syncytial Virus in adults hospitalized with acute respiratory illness may double case detection. Open Forum Infectious Diseases, 9(Supplement_2), 2022.

4. Hirve S, Crawford N, Palekar R, Zhang W. Clinical characteristics, predictors, and performance of case definition-Interim results from the WHO global respiratory syncytial virus surveillance pilot. Influenza Other Respir Viruses, 2020; 14(6):647-657.

5. Papi A, Ison MG, Langley JM, Lee DG, Leroux-Roels I, Martinon-Torres F, et al. Respiratory Syncytial Virus Prefusion F Protein Vaccine in Older Adults. N Engl J Med, 2023; 388(7):595-608.

6. Walsh EE, Pérez Marc G, Zareba AM, Falsey AR, Jiang Q, Patton M, et al. Efficacy and Safety of a Bivalent RSV Prefusion F Vaccine in Older Adults. N Engl J Med, 2023; 388(16):1465-1477.

7. Beran J, Ramirez Villaescusa A, Devadiga R, Nguyen TL, Gruselle O, Pirçon JY, Struyf F, Devaster JM. Respiratory syncytial virus acute respiratory infections in ≥ 65-year-old adults in long-term care facilities in the Czech Republic. Cent Eur J Public Health, 2021; 29(3):167-176.

8. Rubin D. Multiple imputation for nonresponse in surveys. Wiley, 1987.

9. Sharp A, Minaji M, Panagiotopoulos N, Reeves R, Charlett A, Pebody R. Estimating the burden of adult hospital admissions due to RSV and other respiratory pathogens in England. Influenza Other Respir Viruses, 2022; 16(1):125-131.

10. Jansen AG, Sanders EA, Hoes AW, van Loon AM, Hak E. Influenza- and respiratory syncytial virus-associated mortality and hospitalisations. Eur Respir J, 2007; 30(6):1158-1166.

11. Mangtani P, Hajat S, Kovats S, Wilkinson P, Armstrong B. The association of respiratory syncytial virus infection and influenza with emergency admissions for respiratory disease in London: an analysis of routine surveillance data. Clin Infect Dis, 2006; 42(5):640-646.

12. Auvinen R, Syrjänen R, Ollgren J, Nohynek H, Skogberg K. Clinical characteristics and population-based attack rates of respiratory syncytial virus versus influenza hospitalizations among adults-An observational study. Influenza Other Respir Viruses, 2022; 16(2):276-288.

13. Fleming DM, Taylor RJ, Lustig RL, Schuck-Paim C, Haguinet F, Webb DJ, Logie J, Matias G, Taylor S. Modelling estimates of the burden of Respiratory Syncytial virus infection in adults and the elderly in the United Kingdom. BMC Infect Dis, 2015; 15:443.

14. Surveillance data in Denmark, 2023. <https://experience.arcgis.com/experience/220fef27d07d438889d651cc2e00076c/page/RS-virus/>. Accessed 11 Dec 2023.

15. Chorazka M, Flury D, Herzog K, Albrich WC, Vuichard-Gysin D. Clinical outcomes of adults hospitalized for laboratory confirmed respiratory syncytial virus or influenza virus infection. PLoS One, 2021; 16(7):e0253161.

16. Boattini M, Almeida A, Christaki E, Marques TM, Tosatto V, Bianco G, et al. Severity of RSV infection in Southern European elderly patients during two consecutive winter seasons (2017-2018). J Med Virol, 2021; 93(8):5152-5157.

17. Heppe-Montero M, Gil-Prieto R, Del Diego Salas J, Hernández-Barrera V, Gil-de-Miguel Á. Impact of Respiratory Syncytial Virus and Influenza Virus Infection in the Adult Population in Spain between 2012 and 2020. Int J Environ Res Public Health, 2022; 19(22).

18. Niekler P, Goettler D, Liese JG, Streng A. Hospitalizations due to respiratory syncytial virus (RSV) infections in Germany: a nationwide clinical and direct cost data analysis (2010-2019). Infection, 2023.

19. Loubet P, Fernandes J, de Pouvourville G, Sosnowiez K, Elong A, Guilmet C, et al. Respiratory syncytial virus-related hospital stays in adults in France from 2012 to 2021: A national hospital database study. J Clin Virol, 2024; 171:105635.

20. Haeberer M, Bruyndonckx R, Polkowska-Kramek A, Torres A, Liang C, Nuttens C, et al. Estimated Respiratory Syncytial Virus-Related Hospitalizations and Deaths Among Children and Adults in Spain, 2016-2019. Infect Dis Ther, 2024; 13(3):463-480.

21. Polkowska-Kramek A, Begier E, Bruyndonckx R, Liang C, Beese C, Brestrich G, et al. Estimated Incidence of Hospitalizations and Deaths Attributable to Respiratory Syncytial Virus Infections Among Adults in Germany Between 2015 and 2019. Infect Dis Ther, 2024; 13(4):845-860.

22. Liang C, Begier E, Hagel S, Ankert J, Wang L, Schwarz C, et al. Incidence of RSV-related hospitalizations for ARIs, including CAP: Data from the German prospective ThEpiCAP study. J Infect, 2025; 90(3):106440.

23. Osei-Yeboah R, Johannesen CK, Egeskov-Cavling AM, Chen J, Lehtonen T, Fornes AU, et al. Respiratory Syncytial Virus-Associated Hospitalization in Adults With Comorbidities in 2 European Countries: A Modeling Study. J Infect Dis, 2024; 229(Supplement_1):S70-s77.

24. Méroc E, Liang C, Iantomasi R, Onwuchekwa C, Innocenti GP, d’Angela D, et al. A Model-Based Estimation of RSV-Attributable Incidence of Hospitalizations and Deaths in Italy Between 2015 and 2019. Infectious Diseases and Therapy, 2024; 13(11):2319-2332.
